# Design of a novel EmTSP-3 and EmTIP based multi-epitope vaccine against *Echinococcus multilocularis* infection

**DOI:** 10.3389/fimmu.2024.1425603

**Published:** 2024-09-16

**Authors:** Yichen Fan, Yueyue He, Yujiao Li, Zhengwei Yin, Juan Shi, Tingting Tian, Kaiyu Shang, Huidong Shi, Fengbo Zhang, Hao Wen

**Affiliations:** ^1^ State Key Laboratory of Pathogenesis, Prevention and Treatment of High Incidence Diseases in Central Asia, Clinical Medicine Institute, The First Affiliated Hospital of Xinjiang Medical University, Urumqi, China; ^2^ Department of Immunology, School of Basic Medical Sciences, Xinjiang Medical University, Urumqi, China; ^3^ Department of Blood Transfusion, The First Affiliated Hospital of Xinjiang Medical University, Urumqi, China; ^4^ Department of Clinical Laboratory, The First Affiliated Hospital of Xinjiang Medical University, Urumqi, China

**Keywords:** *Echinococcus multilocularis*, immunoinformatics, vaccine, molecular docking simulation, multi-epitope vaccine

## Abstract

**Background:**

Current treatments and prevention strategies for echinococcosis are inadequate. Recent advancements in molecular vaccine development show promise against *Echinococcus granulosus*; however, *Echinococcus multilocularis* remains a challenge. A Multi-epitope Vaccine could potentially induce specific B and T lymphocyte responses, thereby offering protection against *Echinococcus multilocularis* infection.

**Methods:**

This study aimed to develop a MEV against alveolar echinococcosis. Key epitopes from the *Echinococcus multilocularis* proteins EmTSP3 and EmTIP were identified using immunoinformatics analyses. These analyses were conducted to assess the MEV feasibility, structural characteristics, molecular docking, molecular dynamics simulations, and immune simulations. The immunogenicity and antigenicity of the vaccine were evaluated through *in vitro* and *in vivo* experiments, employing ELISA, Western blotting, FCM, challenge infection experiments, and ELISPOT.

**Results:**

The effective antigenicity and immunogenicity of MEV were demonstrated through immunoinformatics, as well as *in vitro* and *in vivo* experiments. *In vitro* experiments revealed that MEV increased the secretion of IFN-γ and IL-4 in PBMC and successfully bound to specific antibodies in patient serum. Furthermore, mice immunized with MEV developed a robust immune response, characterized by elevated levels of CD4+ and CD8+ T-cells, increased secretion of IFN-γ and IL-4 by specific Th1 and Th2 cells, and heightened serum antibody levels. Importantly, MEV reduced the weight of cysts by conferring resistance against echinococcosis. These findings suggest that MEV is a promising candidate for the prevention of *Echinococcus multilocularis* infection.

**Conclusion:**

A total of 7 CTL, 7 HTL, 5 linear B-cell, and 2 conformational B-cell epitopes were identified. The vaccine has demonstrated effective antigenicity and immunogenicity against AE through molecular docking, immune simulation, molecular dynamics studies, and both *in vitro* and *in vivo* experiments. It provides effective protection against *Echinococcus multilocularis* infection, thereby laying a foundation for further development.

## Introduction

Echinococcosis, which includes cystic echinococcosis (CE) caused by *Echinococcus granulosus* and alveolar echinococcosis (AE) caused by *Echinococcus multilocularis* (*E. multilocularis*), is a significant global health concern (1). However, the current diagnostic methods have limitations, and the available drugs are both toxic and ineffective, posing challenges for surgical interventions and overall disease management (2). Consequently, there is an urgent demand for improved diagnostic tools, the discovery of new drug and vaccine targets, and the development of effective preventative strategies, with vaccine development being a critical approach (3).

CE involves dogs or other canids as definitive hosts and ungulates or humans as intermediate hosts, leading to the formation of slow-growing, fluid-filled cysts in the liver and lungs of humans (4). AE is associated with foxes, dogs, and cats as definitive hosts, with humans also being intermediate hosts. In humans, AE results in the development of aggressive, tumor-like lesions that invade tissues and can be fatal if not treated (2). While progress has been made in the development of the EG95 vaccine for CE, research on vaccines for AE remains significantly behind (5). Given the seriousness and potential lethality of AE, there is a compelling need to intensify efforts toward the design of an effective vaccine for its prevention.

MEVs have the advantage over traditional vaccines in selectively and accurately stimulating immune responses without the need for *ex vivo* cultivation, leading to improved biosafety. MEVs are known for their enhanced immunogenicity and broad protective efficacy (6, 7). This approach is especially important in addressing echinococcosis due to the complex life cycle and immune evasion strategies of *E. multilocularis* (8). With the rapid progress in molecular biology, the abundance of genomic and proteomic databases now offers new targets for vaccine development. In recent years, the leucine zipper-like (LEL) domain of tetraspanins (TSP) has garnered attention for its crucial role in mediating protein-protein interactions and homotypic dimerization, primarily attributed to its highly conserved extracellular large loop (9). The interactions between hosts and parasites, believed to be associated with immune evasion, have underscored TSP as promising vaccine candidates to combat the survival mechanisms of schistosomes (10). Given the widespread presence of TSP in various organisms, targeting these proteins could potentially provide broad-spectrum activity against a range of parasites beyond just schistosomes. A recent study have assessed the vaccine efficacy of seven *E. multilocularis* tetraspanins (EmTSP1-7) against AE (10). Remarkably, the recombinant forms of EmTSP3 (rEmTSP3) have resulted in over an 85% reduction in liver cyst lesion numbers (CLNR), indicating their therapeutic potential (9). A recent study has identified a new excretory-secretory product of *E.multilocularis*, known as EmTIP, which shares similarities with immunomodulatory proteins found in humans and mice (11). The research indicates that EmTIP plays a key role in supporting the early growth of the parasite by facilitating interactions between cells and the extracellular matrix (11). This study highlights the importance of the EmTIP homologue in promoting the growth and differentiation of the parasite’s primary cells into metacestode vesicles (11). Additionally, EmTIP has been shown to stimulate the production of interferon-gamma (IFN-γ) by murine CD4+ T-cells in laboratory settings. IFN-γ and its effects are particularly prominent in the initial stages of infection and may have negative impacts on the larval forms of the parasite (11). As metacestodes develop and spread within the host, the Th1 immune response triggered by IFN-γ gradually decreases (11). The discovery and characterization of EmTSP3 and EmTIP not only improve our understanding of host-parasite interactions but also suggest that they could be valuable candidates for the development of new vaccines against AE.

This research employed immunoinformatics to analyze the structural and physicochemical properties of the EmTSP3 and EmTIP proteins. Additionally, it predicted T-cell and B-cell epitopes using various tools to ensure a comprehensive analysis. Effective antigens are characterized by their high antigenicity and immunogenicity, which are critical for eliciting a targeted immune response (12, 13). This study enhances immunogenicity by combining multiple antigen epitopes into a single fusion protein, incorporating epitopes from both EmTSP3 and EmTIP proteins. The research encompasses a comprehensive examination of the physicochemical characteristics of the MEV, a detailed analysis of its tertiary structure, and molecular docking simulations to investigate the interactions between the MEV and Toll-Like Receptor 4 (TLR4). Additionally, molecular dynamics simulations were conducted to further assess the stability and dynamic properties of the docking results. Codon optimization was performed using bioinformatics analyses, and expression validation was executed using simulated PCR and agarose gel electrophoresis. Finally, a series of *in vitro* and *in vivo* experiments were conducted to verify the effectiveness of MEV. The study provides a better understanding of the structural integrity and potential immunological interactions, offering valuable insights for the development of MEV against AE.

## Methods

### The amino acid sequences of proteins

The amino acid sequences of proteins EmTSP3 and EmTIP were obtained from the National Center for Biotechnology Information (NCBI) online database (https://www.ncbi.nlm.nih.gov/). The version of Protein EmTSP3 was ACJ02404.1 and accession was ACJ02404. The version of Protein EmTIP was CCV01194.1 and accession was CCV01194.

### The prediction of target proteins

The physicochemical parameters of proteins EmTSP3 and EmTIP were analyzed from the online software ProtParam (http://web.expasy.org/protparam/), including atomic composition, molecular weight, theoretical isoelectric point (PI), charged polar, stability, hydrophobicity and so on. The transmembrane domains of proteins EmTSP3 and EmTIP were analyzed from the online server TMHMM (https://services.healthtech.dtu.dk/services/TMHMM-2.0/) (14). The signal peptide of proteins EmTSP3 and EmTIP were analyzed from the online server SignalP-5.0 (https://services.healthtech.dtu.dk/services/SignalP-5.0/) (15).

The phosphorylation sites of proteins EmTSP3 and EmTIP were analyzed from the online server NetPhos3.1 (https://services.healthtech.dtu.dk/services/NetPhos-3.1/) (16).

The secondary structure of proteins EmTSP3 and EmTIP were analyzed from software DNASTAR and online software SOMPA (https://npsa-prabi.ibcp.fr/cgi-bin/npsa_automat.pl?page=npsa%20_sopma.html), including Alpha helix, Extended strand, Beta turn and Random coil (17).

To ensure the accuracy of our structural predictions, we utilized the SWISS-MODEL platform (https://swissmodel.expasy.org/) to select high homology models of the proteins EmTSP3 and EmTIP. This approach enhanced the reliability of our computational analysis by providing high sequence identity and validating the models with Ramachandran plots (18).

The T-cell antigen receptor (TCR) is a specialized receptor that enables T-cells to recognize antigens, with a primary focus on identifying the major histocompatibility complex (MHC) types displayed on antigen-presenting cells (APC). T-cell epitopes are categorized into CD8+ and CD4+ groups, with MHC known as human leukocyte antigen (HLA) in the human immune system. Research conducted in Xinjiang, China highlighted prevalent HLA alleles such as HLA-A*11:01 (13.46%), HLA-A*02:01 (12.50%), HLA-A*03:01 (10.10%), HLA-DRB1*07:01 (16.35%), HLA-DRB1*15:01 (8.65%), and HLA-DRB1*03:01 (7.69%) (19). To analyze these T-cell epitopes, we employed the Immune Epitope Database (IEDB) (http://tools.iedb.org/mhci/), NetCTLpan-1.1 (https://services.healthtech.dtu.dk/services/NetCTLpan-1.1/) and NetMHCIIpan-4.0 (https://services.healthtech.dtu.dk/services/NetMHCIIpan-4.0/) databases (20). The selection of T-cell epitopes was based on integrating results from multiple software tools to enhance accuracy. High-scoring predictions from these tools guided the selection of the top 10 or 19-20 epitopes from each software. Antigenicity assessment was performed using the VaxiJen v2.0 platform (https://www.ddg-pharmfac.net/vaxijen/VaxiJen/VaxiJen.html), allergenic potential was determined through the AllergenFP v1.0 platform (https://ddg-pharmfac.net/AllergenFP/feedback.py), and epitope toxicity was evaluated using the ToxinPred platform (https://webs.iiitd.edu.in/raghava/toxinpred/design.php) (21–23). Ultimately, we identified 7 CTL and 7 HTL dominant epitopes, characterized by effective antigenicity while being free from toxic and allergenic properties.

**Figure 1:**
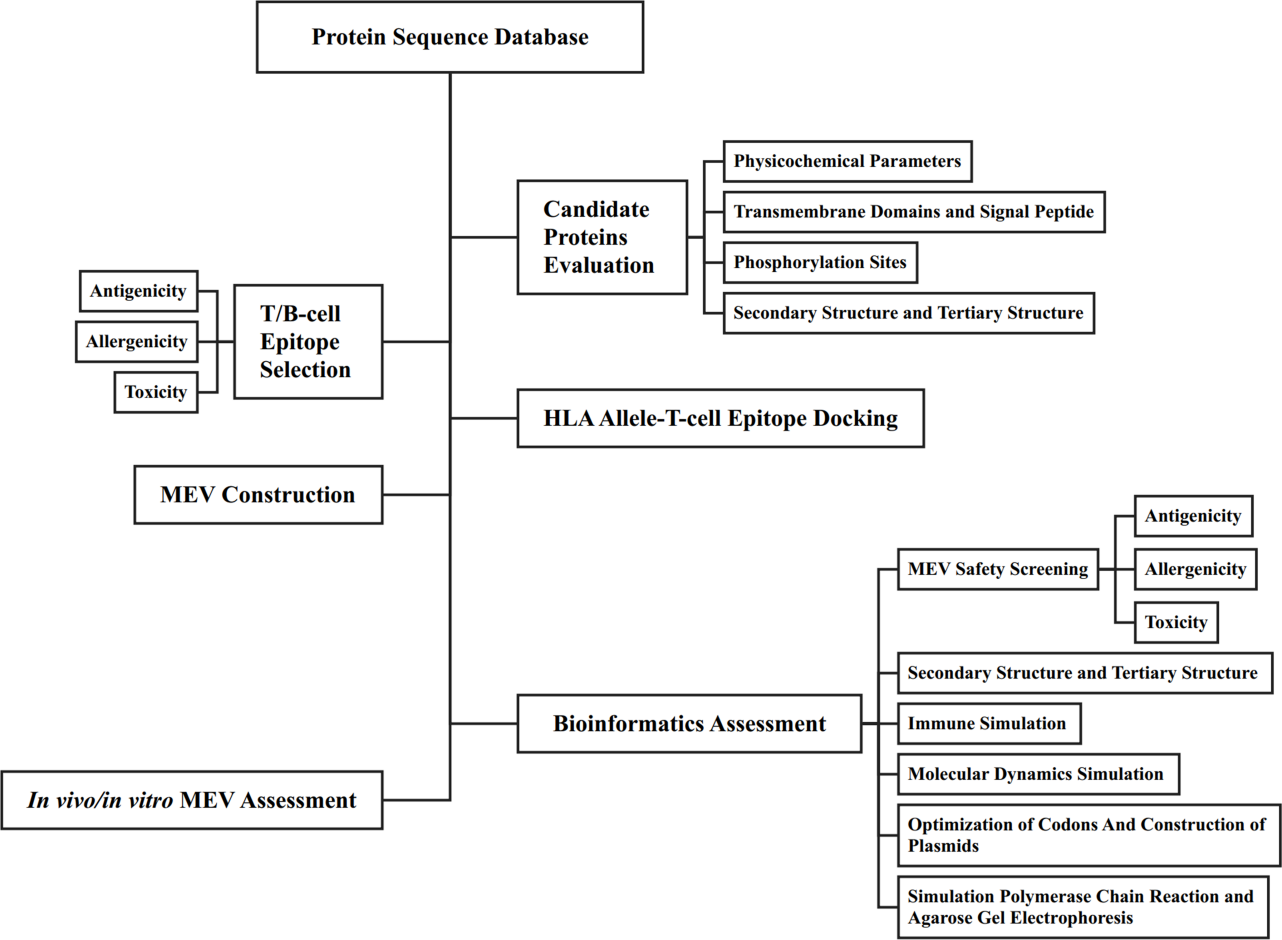
The flow of vaccine design and evaluation.

The primary function of B-cell epitopes is to induce antibody production by recognizing specific antigenic sites. These epitopes can be linear sequences of consecutive amino acid residues on the protein surface or conformational sites formed by spatially adjacent residues in the protein’s three-dimensional structure. B-cell epitopes analysis of EmTSP3 and EmTIP was conducted using the IEDB (http://tools.immuneepitope.org/main/bcell/), with a focus on surface accessibility, flexibility, antigenicity, and hydrophilicity. These parameters were subsequently evaluated using DNASTAR software to identify potential epitope regions. The flowchart outlines the workflow for designing a MEV, encompassing both bioinformatic assessment and *in vitro*/*in vivo* experimental evaluation (**Figure 1**).

### The molecular docking between HLA alleles and T-cell epitopes

To explore the molecular interactions between HLA alleles and T-cell epitopes, we specifically chose HLA class I (HLA-A*02:01) and HLA class II (HLA-DRB1*01:01) alleles for molecular docking. Utilizing the HDOCK server (http://hdock.phys.hust.edu.cn/), our analyses aimed to reveal the essential structural connections important for T-cell recognition and modulation of immune responses (24).

### The construction of MEV

In the construction of the MEV, epitopes from proteins EmTSP3 and EmTIP were connected via some linkers. The EAAAK linker contributes to maintaining protein stability and enhancing its antigenicity (25). Beta-defensin-3 (accession: Q5U7J2) is a naturally occurring antimicrobial peptide with notable immunomodulatory properties. It not only directly eliminates pathogens but also activates and modulates the immune system to bolster the host’s immune defenses (26). PADRE (sequence: AKFVAAWTLKAAA) is a versatile HLA-DR binding epitope capable of binding to various HLA-DR molecules, thereby facilitating antigen presentation and enhancing immune responses in individuals with diverse HLA types (27). To ensure optimal presentation and functionality of individual epitopes within the vaccine, specific linking strategies were employed. B-cell epitopes were linked with a double lysine (KK) linker to enhance flexibility and antigen presentation efficiency (28). HTL epitopes were connected using a GS linker to improve their display and immune response, while CTL epitopes were linked with an AAY linker to optimize their functionality within the vaccine (29). RVVR linker is a flexible and hydrophilic linker that helps to improve the solubility and stability of the MEV (30). Furthermore, a His-tag was added at the C-terminus of the vaccine to aid in purification and detection processes (31).

### The evaluation of vaccine construct

Following the construction of vaccine, the allergenicity, antigenicity and toxicity of vaccine needs to be evaluated for using the AllergenFP v1.0 platform, VaxiJen v2.0 platform and ToxinPred platform. The physicochemical properties of the MEV were systematically analyzed using the online software ProtParam. The solubility prediction of the MEV was carried out with SOLpro platform (http://scratch.proteomics.ics.uci.edu/). The analysis of MEV and human homology was conducted using the Basic Local Alignment Search Tool (BLAST) from the NCBI (https://blast.ncbi.nlm.nih.gov/Blast.cgi) (32).

### The secondary and tertiary structure of vaccine

The secondary structure of MEV was analyzed from the online software SOMPA. The tertiary structure was constructed from the online platform AlphaFold v2.3.0 (33). By incorporating multi-sequence alignments into its algorithmic design, AlphaFold utilizes deep learning techniques to achieve remarkable predictive accuracy in protein structure determination. After construction, use GalaxyWEB server (https://galaxy.seoklab.org/cgi-bin/submit.cgi?type=REFINE) to refine it again (34). Quality verification of vaccine’s tertiary structure models was conducted using ERRAT (https://saves.mbi.ucla.edu/) and ProSA-web (https://prosa.services.came.sbg.ac.at/prosa.php/) and PDBsum (https://www.ebi.ac.uk/thornton-srv/databases/pdbsum/Generate.html) server focusing on non-bonded interactions, Ramachandran plot observations and Z-score values (35).

### The molecular docking between TLR4 and MEV

In order to explore the molecular mechanism of vaccine-induced immune response, we used molecular docking technology to simulate the binding process of vaccine proteins and immune molecules. Docking simulations were performed using the HDOCK server (http://hdock.phys.hust.edu.cn/) to analyze the interaction between the vaccine and the TLR4 immune receptor (PDB ID: 4G8A). By examining receptor-ligand interactions through 2D diagrams generated by Discovery Studio software, we identified the critical interacting residues. Furthermore, the 3D structure of these interactions was visualized using PyMOL software.

### The molecular dynamics simulation

Molecular dynamics simulation was carried out using Gromacs2022.3 software (29). Small molecules were prepared with AmberTools22 to add the GAFF force field, hydrogenate them, and calculate RESP potentials using Gaussian 16W. This potential data was then integrated into the topology file of the molecular dynamics system. The simulation was conducted under constant temperature (300K) and atmospheric pressure (1 Bar), with the Amber99sb-ildn force field. Water molecules were used as the solvent with the Tip3p water model, and neutrality was achieved by adding an appropriate number of Na+ ions. Energy minimization was done through the steepest descent method, followed by equilibration in both the NVT and NPT ensembles for 100,000 steps each, with a coupling constant of 0.1 ps and a duration of 100 ps. Subsequently, free molecular dynamics simulation was carried out for 5,000,000 steps with a step length of 2 fs, totaling 100 ns. After the simulation, a comprehensive analysis was performed using the software’s tools to calculate RMSD, RMSF, protein rotation radius for each amino acid trajectory, as well as MMGBSA free energy, free energy topography, and other relevant data (30).

### The immune simulation

The MEV, upon entry, acts as an antigen triggering immune activation. We utilized the C-ImmSim platform (https://kraken.iac.rm.cnr.it/C-IMMSIM/index.php?page=1) to simulate the immune responses induced by the vaccine. Our focus was on the prevalent HLA alleles in the Xinjiang population, which include HLA-A*1101, HLA-A*0201, HLA-A*0301, HLA-DRB1*0301, HLA-DRB1*0701, and HLA-DRB1*1501 (36). The simulation encompassed three anatomical regions (bone marrow, thymus, lymph nodes) across intervals of 1, 84, and 168 days. We used default settings, a parameter random seed of 12,345, a volume of 50, and 1050 steps.

### The optimization of codons and construction of plasmids

The codons of the vaccine were optimized using the JAVA codon adaptation tool (http://www.jcat.de/) after selecting XHOI and BamHI restriction endonuclease sites (37). The DNA sequence of the plasmid was obtained, with pET28a (+) chosen as the vector. Subsequently, the endonuclease sites within the multiple cloning site (MCS) region were analyzed using SnapGene 6.0.2 software to insert the vaccine target gene sequence into the plasmid, completing the in silico cloning process.

### The simulation polymerase chain reaction and agarose gel electrophoresis

In SnapGene 6.0.2, primers were designed with consideration for the Tm value and length, typically ranging from 15 to 30 base pairs with a selected Tm value of 72°C. The annealing temperatures of the upstream and downstream primers differ by 1°C, and both have a GC content maintained at approximately 40–60%, with a protective nucleobase added at the 5’ end. Finally, the recombinant plasmid underwent simulated agarose gel electrophoresis in SnapGene 6.0.2.

### Patients

In this study, we included 46 patients (adults aged 18–60) diagnosed with AE from the Infection Department of the First Affiliated Hospital of Xinjiang Medical University. Additionally, we enrolled 12 healthy individuals (adults aged 18–60). All selected patients met the Chinese guidelines for the diagnosis and treatment of AE, and any other conditions that could potentially affect the experimental results were excluded. Peripheral blood (4 ml per person) was collected from both patients and healthy individuals, and serum was subsequently separated for Western blotting analysis. Peripheral blood mononuclear cells (PBMCs) from patients were isolated using Ficoll Paque, and *in vitro* cell experiments were conducted.

### Detecting the immunogenicity of MEV

The ELISA was conducted to evaluate the immunogenicity of MEV. PBMCs were isolated and collected from patients diagnosed with AE. These cells were then stimulated for 48 hours in a 37°C incubator with 5% CO2 using either aseptic PBS buffer (Control group, n=6) or MEV protein (MEV group, n=6). After stimulation, the cell culture supernatant was harvested for the detection of IFN-γ and IL-4 via ELISA. The culture supernatant was distributed into separate ELISA plates designated for IFN-γ and IL-4, with two replicate wells established for each sample. All subsequent procedures were performed in strict accordance with the manufacturer’s instructions. The Human IFN-γ ELISA Kit (SEKH-0046, Solarbio Science & Technology Co., Ltd, Beijing, China) and the Human IL-4 ELISA Kit (SEKH-0011, Solarbio Science & Technology Co., Ltd, Beijing, China) were utilized.

### Detecting the antigenicity of MEV

The Western Blot experiment was conducted to detect the antigenicity of MEV. The loading buffer, containing MEV at a concentration of 1 µg/µL, was subjected to SDS-PAGE, followed by the transfer of proteins to a PVDF membrane. After blocking with a 5% skimmed milk powder solution, the PVDF membrane was incubated overnight at 4°C in human serum (1:200 dilution) from both AE patients and healthy individuals. Subsequently, the membrane was treated with a rabbit anti-human IgG secondary antibody labeled with HRP (1:2000 dilution) at room temperature for 1 hour. Finally, the target protein bands were visualized using an ECL detection kit (BL523B, BioSharp, Beijing, China) and exposed on Amersham HyperFilm (GE Healthcare).

### Animals

In this research, 8-week-old SPF-grade BALB/C mice were obtained from the Animal Experiment Center at Xinjiang Medical University. The mice were randomly divided into two groups: the MEV group (n=12), which received an intraperitoneal injection of MEV mixed with an equal volume of Freund’s adjuvant (50 ug/mouse), and the Control group (n=12), which received PBS mixed with an equal volume of Freund’s adjuvant (50 ug/mouse). Immunizations were performed biweekly for a total of three times. The first immunization employed complete Freund’s adjuvant (CFA), while the second and third booster immunizations utilized incomplete Freund’s adjuvant (IFA). Blood samples were collected from the inner canthus of the eye prior to each immunization to monitor the dynamic changes in antibody levels. Two weeks after the final immunization, six mice from each group were euthanized, and their serum and splenocytes were collected for FCM, ELISA, and ELISPOT. Additionally, a potential protection assay was conducted on six mice from both the vaccinated and Control groups to determine whether MEV could induce resistance in the host against infection by the original head segment.

### Detecting the immune response of mouse T-cell

#### Flow cytometry experiment

Two weeks following the final immunization, splenocytes were harvested from the mice. Lymphocytes were counted using a hemocytometer, and a splenocyte suspension was prepared with a cell concentration adjusted to 2 × 10^6 cells/mL. The splenocyte suspension was subsequently incubated with anti-CD3 (APC), anti-CD8 (PE-Cy5), and anti-CD4 (FITC) monoclonal antibodies for 20 minutes. FCM was then performed to identify CD4+ T-cells by gating on CD3+CD4+ cells, and to identify CD8+ T-cells by gating on CD3+CD8+ cells using FlowJo software. The flow cytometer utilized in this study was the BeamCyte-1026-0097.

#### Enzyme-linked immunospot test

To further evaluate the HTL response, we analyzed the levels of IFN-γ and IL-4 in immunized mice using an ELISPOT. ELISPOT plates were coated overnight at 4°C with anti-mouse IFN-γ and IL-4 capture antibodies (BD PharMigen, San Diego, CA) diluted 1:60 in PBS. Following this, the plates were washed with PBS and incubated with blocking buffer (1% BSA) at room temperature for 1 to 2 hours. A total of 100 μL of splenocyte suspension was added to each well, with MEV (10 μg/mL) protein introduced to both the MEV and Control groups, along with synthetic Toxoplasma SAG4 peptide (10 μg/mL). ConA (10 μg/mL) at 50 μL/well and PBS at 50 μL/well served as positive and negative controls, respectively. The plates were then incubated for 24 hours at 37°C in an atmosphere containing 5% CO2. After washing, 50 μL of biotin-labeled secondary antibody (BD PharMigen, San Diego, CA) was added, and the plates were incubated at room temperature for 2 hours. Following another wash, 50 μL of streptavidin alkaline phosphatase (Amersham Life Science, Australia) was added, and the plates were incubated at room temperature for an additional 2 hours. The development of spots was achieved by adding BCIP/NBT developer (Moss, Inc, Pasadena, MD, USA) to each well for a 40-minute incubation at room temperature. Finally, the plates were washed with water to halt the reaction, and spot-forming units (SFU) in each well were counted using an ELISPOT automatic tablet reader (AID EliSPOT Reader, Germany). The results were expressed as IFN-γ and IL-4 spot-forming cells (SFC) per million cells.

### Detecting the level of specific antibody

To further evaluate the antigenicity of MEV, an ELISA was employed to measure the levels of anti-MEV-specific antibodies produced in the mouse model. Microplates were coated with 6 μg/ml MEV protein using bicarbonate buffer (pH 9.6), followed by blocking with 5% milk in neutral phosphate-buffered saline containing 0.3% Tween-20 (PBST). Mouse serum samples (100 μl), diluted 1:100 in PBST, were added and incubated for 30 minutes at 37°C. After washing with PBST, an HRP-labeled secondary antibody (1:1000) was added. Finally, absorbance values at a wavelength of 450 nm were measured using a microplate reader.

### Detection of the weight of hydatid cyst

To evaluate the potential protective effects of the designed MEV, which could induce host resistance to a challenge infection with *E. multilocularis*, all mice in the infection model from both the control groups and the MEV group were dissected. The weight of the *E. multilocularis* cysts was measured after 23 weeks of protoscolex infection.

## Results

### The prediction of target proteins

Protein EmTSP3 is composed of 148 amino acids, totaling 2304 atoms, with a chemical formula of C_724_H_1176_N_184_O_206_S_14_ and a molecular weight of 16 kDa (**Supplementary Table 1-1**). The PI is 6.70. This protein contains 15 acidic amino acids (Asp + Glu) and 15 basic amino acids (Arg + Lys). The instability index (II) is calculated to be 31.84, indicating stability (stability is confirmed when II < 40). The aliphatic index is 111.96, and the Grand Average of Hydropathicity (GRAVY) is 0.389, indicating that EmTSP3 has a certain degree of hydrophobicity. Protein EmTIP is comprised of 592 amino acids, totaling 8992 atoms, with a chemical formula of C_2886_H_4474_N_748_O_861_S_23_ and a molecular weight of 64 kDa (**Supplementary Table 1-1**). The PI is 5.11. It contains 57 acidic amino acids (Asp + Glu) and 44 basic amino acids (Arg + Lys). Despite an II of 40.39, slightly above the stability threshold of 40, indicating potential instability, the protein remains close to the boundary between stability and instability. Given this minor deviation, EmTIP could still be a suitable candidate for multi-epitope vaccine design. The aliphatic index is 91.10, and the GRAVY is 0.089, indicating a certain degree of hydrophobicity. The GRAVY values of both proteins suggest a slight tendency towards hydrophobicity, striking a balance between hydrophobicity and hydrophilicity. This moderate hydrophobicity may aid in proper protein folding, functionality, and immune system recognition without excessive aggregation (38).

The online server TMHMM was used to predict the Transmembrane Domains of protein (**Supplementary Figure 1**). SignalP-5.0 predicted that both Protein EmTSP3 and Protein EmTIP each have a single signal peptide. The signal peptide is located at residues 1-21 for EmTSP3 and 1-20 for EmTIP, with a high probability of occurrence (**Supplementary Figure 2**). Removing the signal peptide can help prevent the protein from being mistakenly targeted to the ER or other organelles, ultimately reducing the likelihood of misfolding and mislocalization, which could impact the accuracy of epitope prediction.

Using the NetPhos 3.1 server analysis, the main phosphorylation sites and their targeting kinases for Protein EmTSP3 and Protein EmTIP were predicted (**Supplementary Figure 3**). The secondary structures of proteins EmTSP3 and EmTIP were determined using the DNAstar software and the SOPMA online server. The results from Chou-Fasman and Gramier-Robson are shown in **Supplementary Figure 4**, while the results from SOPMA are displayed in **Supplementary Figures 5-1**, **5-2**. The combined results of these two prediction methods are summarized in **Supplementary Tables 1-2**, **1-3**. The type and distribution of secondary structures impact the antigenicity of epitopes, with certain secondary structures being more easily recognized and bound by the immune system. The high α-helix content in EmTSP3 suggests the presence of stable and exposed epitopes, while EmTIP is mainly composed of random coil structures, potentially providing flexible and accessible epitopes. These secondary structures identify potentially antigenic regions in the proteins, aiding in the selection of effective epitopes for MEV development. The tertiary structures of protein EmTSP3 and EmTIP were built using SWISS-MODEL, showing sequence identities of 100% and 98.77% with protein models B6VFH5.1.A and A0A068WNA4.1.A respectively, and their rationality was confirmed through Ramachandran plots (**Supplementary Figures 6**). The stability and functionality of tertiary structures also impact the formation and recognition of epitopes, including conformational epitopes.

For the prediction of T-cell epitopes, the MHC restriction was considered essential. Thus, CD8+ and CD4+ T-cell epitopes for proteins EmTSP3 and EmTIP were predicted using the IEDB, NetCTLpan and NetMHCIIpan online tools. To enhance accuracy, results from both software were amalgamated to determine the final T-cell epitopes, detailed in **Supplementary Tables 2-1**, **2-2**, **3-1**, **3-2**, and summarized in **Tables 1-1**, **1-2**.

**Table 1-1:** List of the final selected CTL epitopes.

Protein	Allele	Serials	Sequence	Antigenicity	Allergenicity	Toxicity
EmTSP3	HLA-A*03:01	3-15	LVYRHEFVGLVGK	0.89	Non-Allergen	Non-Toxin
	HLA-A*11:01	56-66	WSKPYPASCCK	0.8	Non-Allergen	Non-Toxin
	HLA-A*02:01	83-97	VAMYEQIKDSSLAFG	0.71	Non-Allergen	Non-Toxin
EmTIP	HLA-A*11:01	205-214	SSYPLPIELK	0.98	Non-Allergen	Non-Toxin
	HLA-A*11:01	382-390	VSTSLYMQK	0.98	Non-Allergen	Non-Toxin
	HLA-A*03:01	515-526	AKLFLQPLYDMK	1.14	Non-Allergen	Non-Toxin
	HLA-A*11:01	41-51	ALYALLAPNAK	0.77	Non-Allergen	Non-Toxin

**Table 1-2:** List of the final selected Th epitopes.

Protein	Allele	Serials	Sequence	Antigenicity	Allergenicity	Toxicity
EmTSP3	HLA-DRB1*07:01	6-20	RHEFVGLVGKEMQRE	1.18	Non-Allergen	Non-Toxin
	HLA-DRB1*03:01	14-28	GKEMQREIKDLTAHG	1.3	Non-Allergen	Non-Toxin
	HLA-DRB1*03:01	18-32	QREIKDLTAHGRNAS	1.39	Non-Allergen	Non-Toxin
	HLA-DRB1*03:01	21-35	IKDLTAHGRNASDPL	1.25	Non-Allergen	Non-Toxin
EmTIP	HLA-DRB1*03:01	550-564	YLEVRSDQKERMQES	1.59	Non-Allergen	Non-Toxin
	HLA-DRB1*03:01	21-35	AFADVNTDRRTDAIV	0.72	Non-Allergen	Non-Toxin
	HLA-DRB1*15:01	300-314	DADLDGYPDLAVGLK	0.7	Non-Allergen	Non-Toxin

The line B-cell epitopes (LBEs) and construct B-cell epitopes (CBEs) of proteins EmTSP3 and EmTIP were predicted using DNASTAR and IEDB software. The differential predictive outcomes of the two software were verified based on surface accessibility, flexibility, antigenicity, and hydrophobicity to obtain the initial epitopes of the proteins (**Supplementary Tables 4-1**, **4-2** and **Supplementary Figures 8-1**, **8-2**). Subsequently, we predicted linear and conformational epitopes using IEDB and assessed their antigenicity, allergenicity, and toxicity using three respective online servers (**Supplementary Tables 4-3**, **4-4**). The results from both software were combined to refine the final predictions of B-cell epitopes, with a conclusive summary in **Tables 2-1**, **2-2**.

**Table 2-1:** List of the final selected LEBs.

Protein	Serials	Sequence	Antigenicity	Allergenicity	Toxicity
TSP3	18-28	SSFSLVASWSD	1.16	Non-Allergen	Non-Toxin
	22-33	GGKNVTRSVGSF	1.6	Non-Allergen	Non-Toxin
	66-82	NAFSLQNFTHSATSLSE	1.21	Non-Allergen	Non-Toxin
TIP	483-495	TQQARKHKLTFIV	1.37	Non-Allergen	Non-Toxin
	553-569	VRSDQKERMQESQRFHF	1.03	Non-Allergen	Non-Toxin

**Table 2-2:** List of the final selected CBEs.

Protein	Serials	Sequence	Antigenicity	Allergenicity	Toxicity
TIP	A:E432, A:Q434, A:G435, A:A436, A:D437, A:G438, A:G439, A:R440, A:F468, A:Y471, A:E473, A:K490, A:T492, A:F493	EQGADGGRFYEKTF	1.83	Non-Allergen	Non-Toxin
TIP	A:P49, A:N50, A:A51, A:K52, A:Q53, A:T54, A:D55, A:G56, A:K57, A:P58	PNAKQTDGKP	1.25	Non-Allergen	Non-Toxin

### The molecular docking between HLA alleles and T-cell epitopes

Analysis of the interaction between HLA-I and CTL epitopes revealed a docking score of -261.59 and a ligand RMSD of 31.11 Å. Similarly, the interaction between HLA-II and HTL epitopes revealed a docking score of -259.53 and a ligand RMSD of 210.24 Å. These results collectively suggest that the docked complexes possess high affinity (**Supplementary Figure 9**). The docking analysis suggests that T-cell epitopes bind strongly to MHC molecules, facilitating their presentation by APCs and triggering specific immune responses.

### The construction of MEV

**Figure 2:**
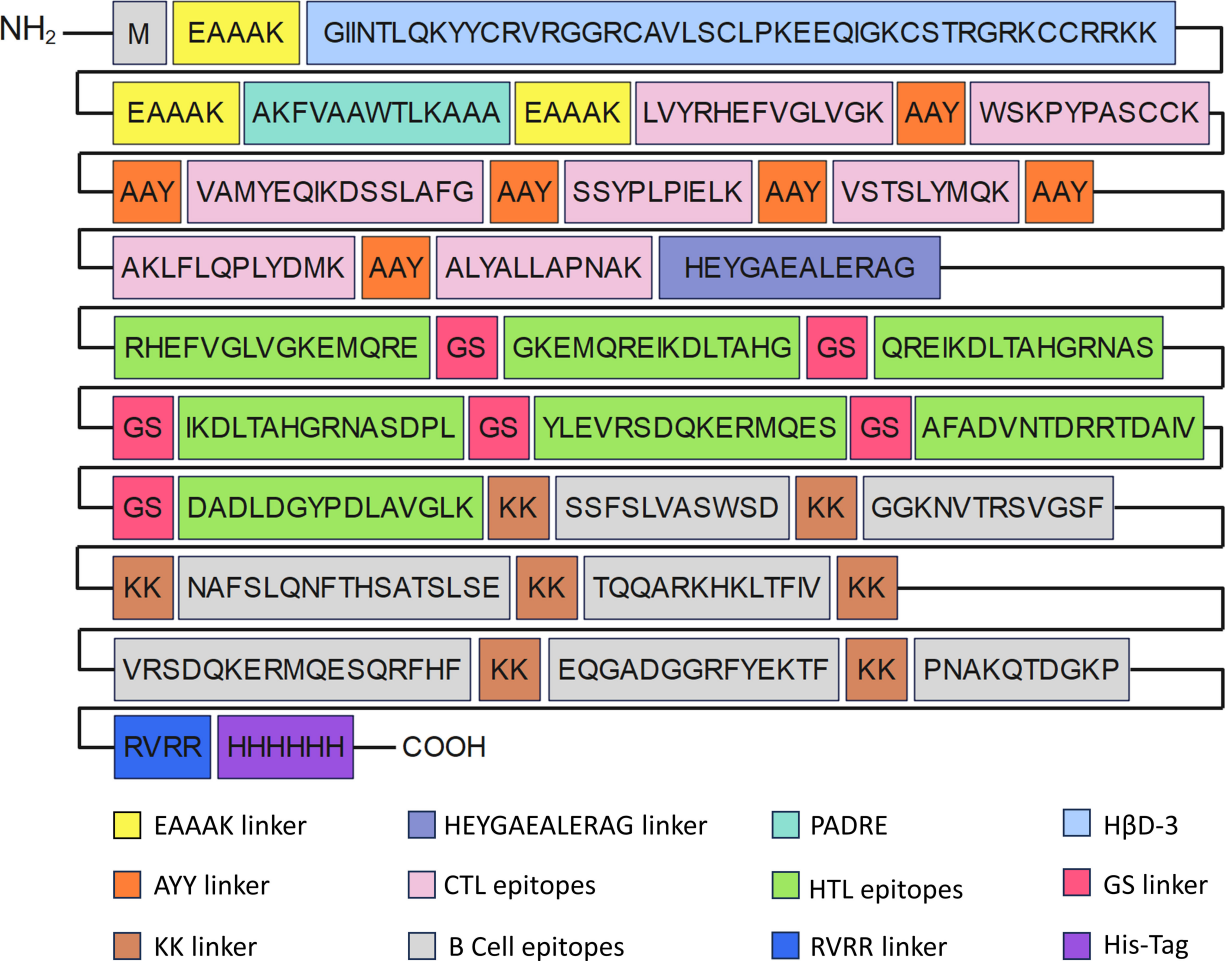
The order of epitopes in construction of MEV.

In the construction of the MEV, epitopes devoid of allergenic and toxic properties were selected. Subsequently, these carefully chosen epitopes were covalently bonded using specific linkers to formulate the intricate vaccine sequence. The construction of the vaccine included dominant epitopes, specifically 7 CTL, 7 Th, 5 LB, and 2 CB epitopes, with a total length of 436 amino acids (**Figure 2**).

### The evaluation of MEV construct

The MEV had a molecular weight of 48.37kDa, and a molecular formula of C_2131_H_3389_N_635_O_624_S_15_. The II was computed to be 27.61, classifying the vaccine as a stable protein. The GRAVY value was -0.662, suggesting that the vaccine is hydrophilic. Furthermore, the vaccine showed an antigenicity of 0.7621, exceeding the threshold, and exhibited no allergenicity. With a solubility of 0.92, the vaccine is considered a soluble protein antigen. The analysis of vaccine and human homology indicates that the obtained sequence has a homology of only 10%, which is insufficient to trigger an autoimmune response (**Supplementary Figure 10**).

### The secondary and tertiary structure of MEV

**Figure 3:**
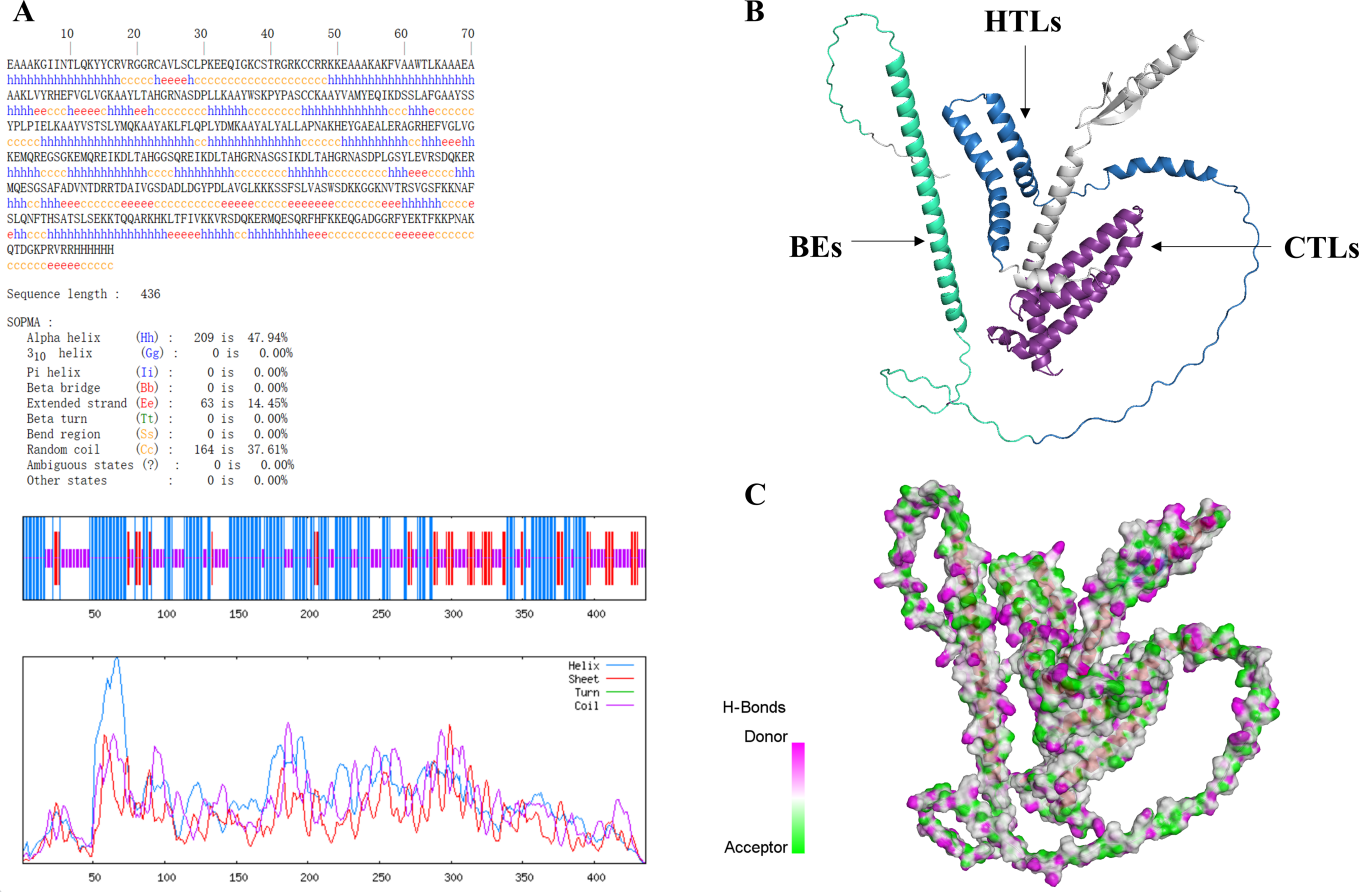
**(A)** Secondary structure prediction results show 47.94% alpha-helix, 37.61% random coil, and 14.45% extended strand. **(B)** In the MEV structure displayed using Discovery Studio. **(C)** Pink regions represent hydrogen bond donors, while green regions represent hydrogen bond acceptors.

The predicted result of secondary structure of MEV by SOMPA was shown in **Figure 3A**. We constructed the prediction model of the MEV tertiary structure using AlphaFold2 server and selected the model with the highest score ranking. Subsequently, we refined the model structure using GalaxyWEB server and ultimately selected the model with the highest GDT-HA score, indicating the best quality assessment of the model (**Figure 3B**). In **Figure 3C**, The donors and acceptors contribute to the stability of the vaccine structure and may play a role in binding to antibodies or T-cell receptors during the immune response.

### The assessment of model quality

**Figure 4:**
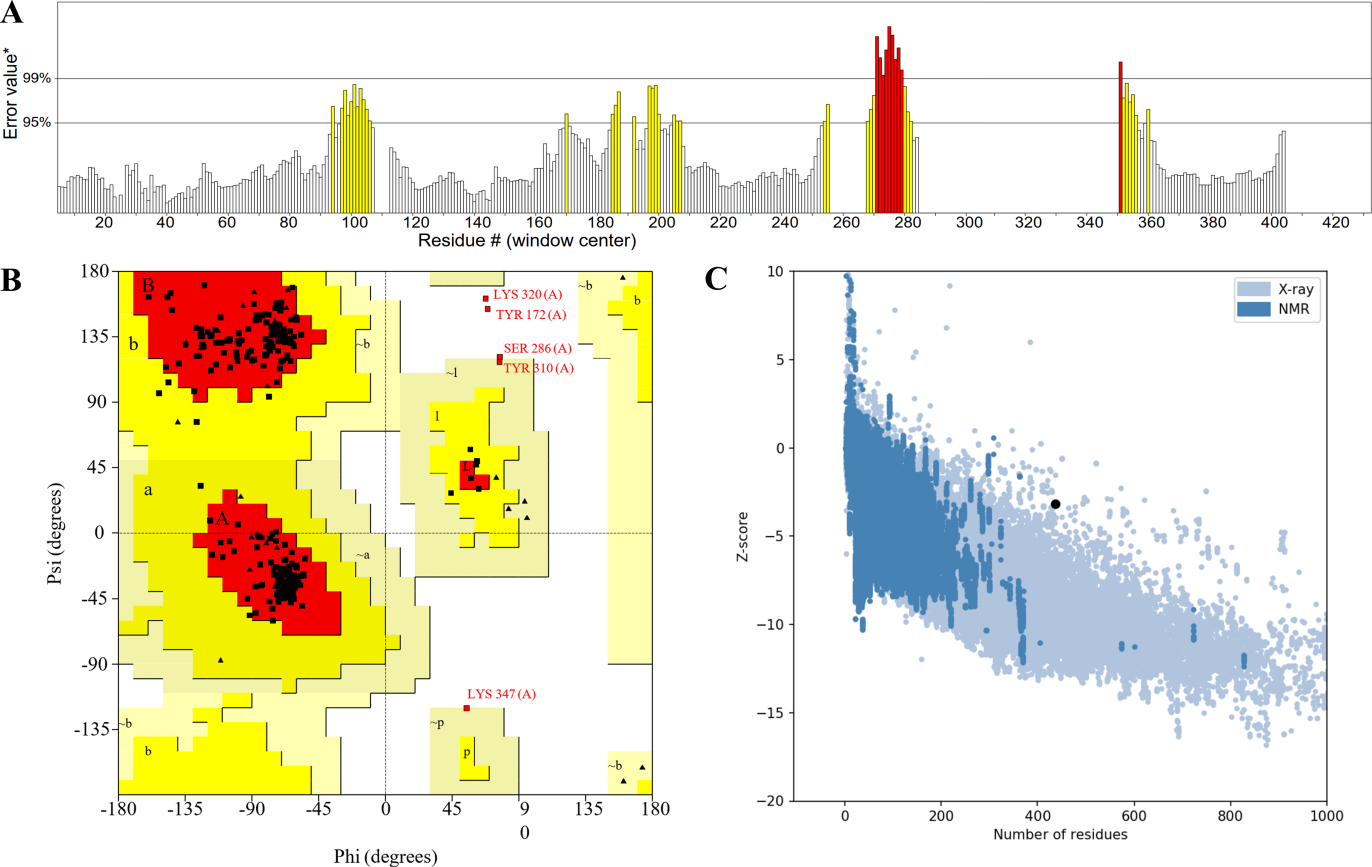
**(A)** The statistics of non-bonded interactions between different atom types. **(B)** The red region signifies the allowed region, the yellow region the maximum allowed region, and the blank region the disallowed region. **(C)** The Z-score plot obtained from ProSA-web, the light blue region represented the structural group analyzed using X-rays, while the dark blue region represented the group analyzed using nuclear magnetic resonance (NMR).

The ERRAT server analyzes the statistics of non-bonded interactions between different atom types and exhibit the result of the error function versus position (**Figure 4A**). A good resolution structure typically produces an overall quality factor of around 91% on average, and we measured an overall quality factor of 84.89%. To validate the structural accuracy, the model’s quality was further assessed using PDBsum structure evaluation service, known as the Ramachandran plots (**Figure 4B**). In the Ramachandran plots, most of the amino acids (represented as dotted structures) are observed to fall within the interval range. ProSA-web server was utilized to assess the quality of the model, and the Z-score obtained from the software reflected the quality of the model (**Figure 4C**). The Z-score for the MEV was -3.17. A Z-score within the range characteristic of native proteins suggests a correctly folded structure. In summary, although the overall quality factor is slightly below the ideal value, multiple independent structural validation methods consistently demonstrate that our MEV tertiary structure model is accurate and reliable.

### The molecular docking between TLR4 and MEV

**Figure 5:**
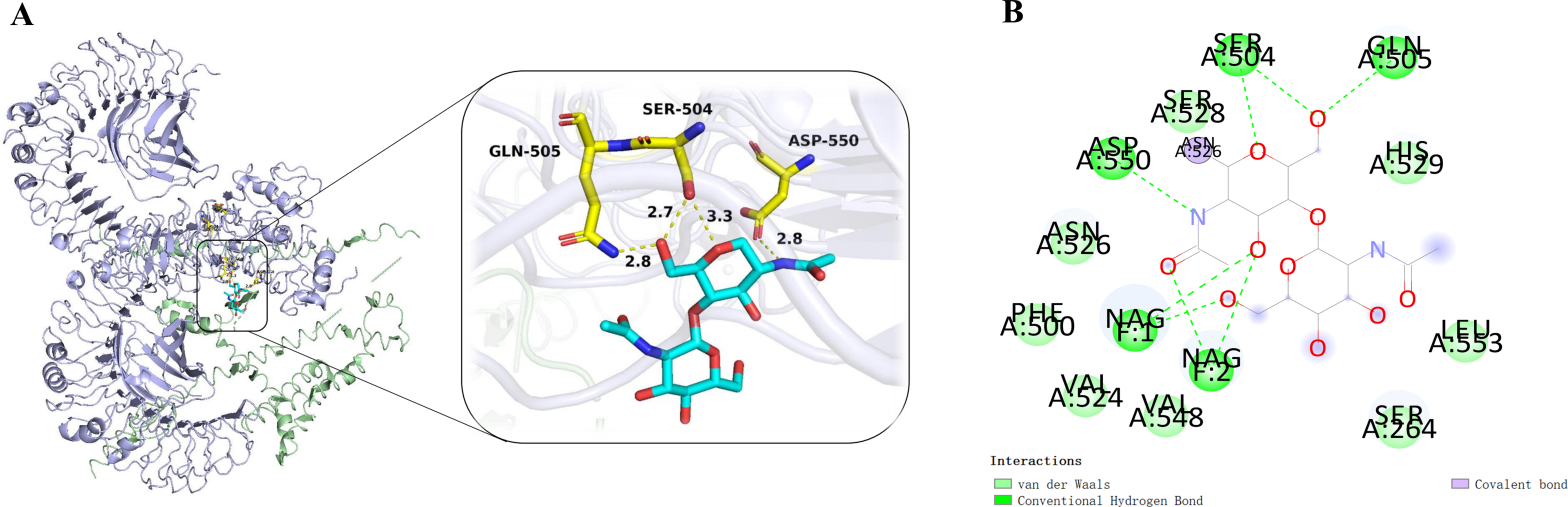
**(A)** The complex of MEV-TLR4 was analyzed for interactions and their 3D images were taken by using visualizing tool PyMol. **(B)** The complex was analyzed for interactions and their 2D images were taken by using visualizing tool Discovery Studio.

Molecular docking utilizes energy minimization and spatial structure complementarity to predict ligand-receptor interactions at the active site. Multiple models were generated, with the top model chosen for analysis. The optimal docking results showed a score of -280.32 and a ligand RMSD of 63.3 Å. The selected docking structure was visualized in 3D using PyMOL (**Figure 5A**), and a 2D interaction map was created with Discovery Studio (**Figure 5B**).

### The molecular dynamics simulation

**Figure 6:**
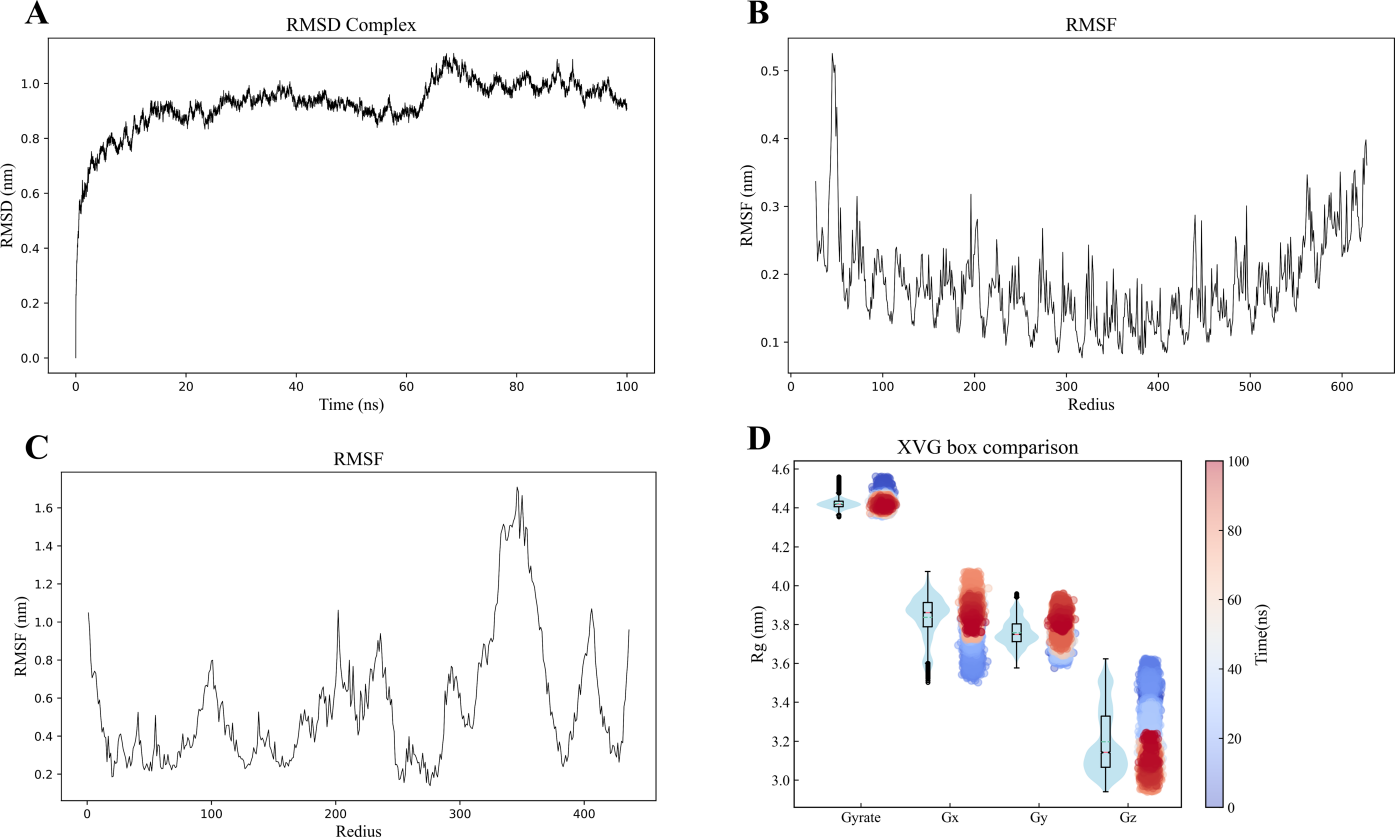
**(A)** Molecular dynamics simulation of the MEV -TLR4 complex. **(B, C)** RMSD of the TLR4 and MEV, respectively. **(D)** The radius of gyration analysis.

The molecular dynamics simulation results demonstrated good structural stability of the MEV-TLR4 complex throughout the simulation. Post-simulation analysis included examining RMSD, RMSF, RoG, and potential energy to assess conformational and structural integrity. The RMSD curve demonstrated stability throughout the simulation, with a slight fluctuation around 65ns, indicating overall structural stability despite transient changes (**Figure 6A**). Similarly, RMSF values for TLR4 and the vaccine proteins exhibited minimal fluctuations, indicating sustained structural integrity (**Figures 6B, C**). The RoG maintained a consistent trend without significant deviations, suggesting that the overall compactness and structural integrity of the complex were maintained during the simulation (**Figure 6D**). The study also utilized RMSD and RoG parameters to display the protein Free Energy Landscape, showing a distinct low-energy region denoted by a deep blue area on the maps, indicating a stable conformation of the protein at 100ns (**Supplementary Figure 11**). The binding free energy calculations indicated a robust and favorable interaction between the vaccine and TLR4, highlighting a strong affinity. Additional energy components, including electrostatic, solvation, and molecular mechanics terms, further characterized the interaction dynamics (**Table 3**).

**Table 3:** Delta G binding free energy.

Energy	Average	Energy	Average
ΔVDWAALS	-351.58	ΔG gas	-6078.65
ΔEEL	-5727.07	ΔG solv	5886.66
ΔEGB	5938.44	ΔTOTAL	-191.98
ΔESURF	-51.77		

*ΔVDWAALS= van der Waals energy; ΔEEL= electrostatic energy; ΔEGB= polar solvation energy; ΔESURF= nonpolar solvation energy; ΔG gas= Molecular mechanics term energy; ΔG solv= solvation energy; ΔTOTAL= ΔGMMGBSA= binding free energy.

### The immune simulation

**Figure 7:**
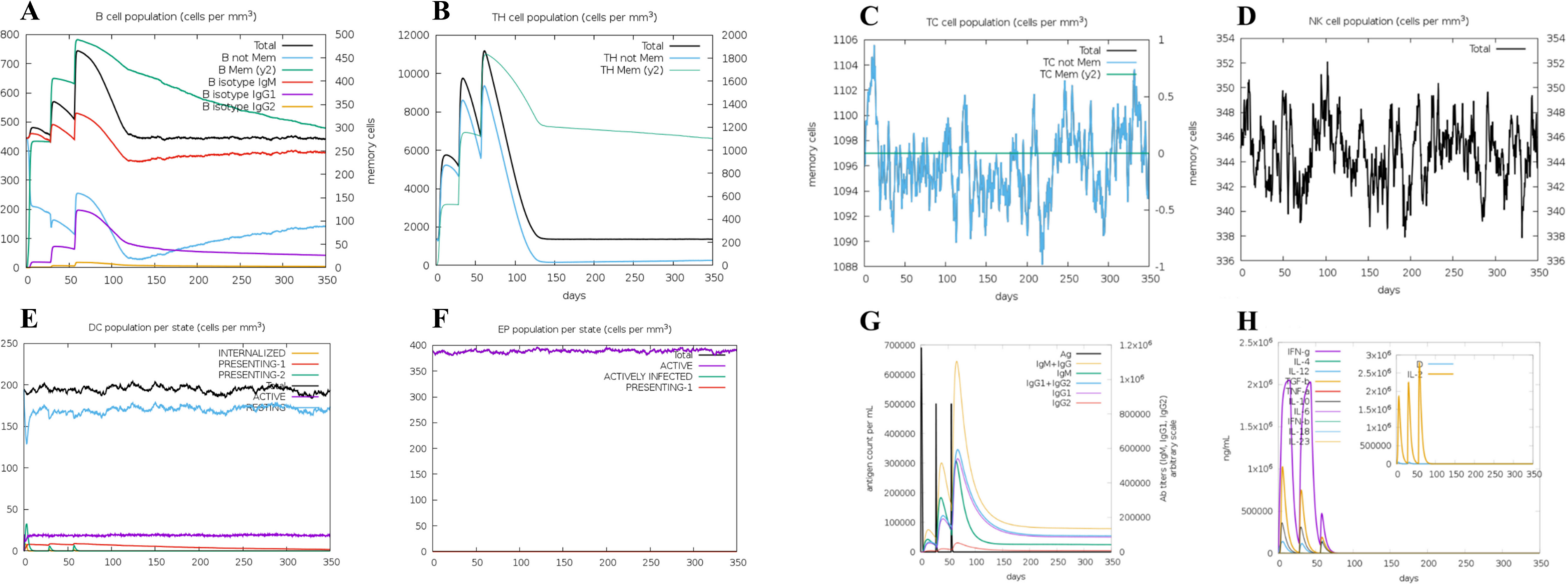
**(A)** The B-cell isotypes. **(B)** The Th-cell isotypes. **(C)** The TC-cell isotypes. **(D)** The NK-cells. **(E)** The DC-cells. **(F)** The EP-cells. **(G)** The immune globulin. **(H)** The interleukins and cytokines.

Utilizing the C-ImmSim server, we employed advanced computational approaches to predict the immune responses stimulated by MEV. Our results reveal a consistent increase in B-cell count, crucial for stimulating immune responses, reaching its peak after the final administration of MEV (**Figure 7A**). Th and cytotoxic T (TC) cells, essential subgroups of T-cells, exhibited a growth trend following three doses of the vaccine, with a peak observed post-inoculation. Conversely, TC cells, NK cells, dendritic cells (DC), and effector memory (EP) cells showed relative stability, yet displayed dynamic changes over time (**Figures 7B–F**). Both primary and secondary immune responses demonstrated a sustained increase in IgM and IgG levels (**Figure 7G**). Remarkably, MEV triggered a robust cytokine response, notably elevating levels of IFN-γ, TGF-β, IL-10, and IL-18 (**Figure 7H**).

### The optimization of codons and construction of plasmids

In this study, codon optimization was meticulously conducted with the aim to enhance the codon adaptation index (CAI), closely approximating the optimal value of 1.00. Notably, the optimized CAI for the MEV sequence reached 0.82. Regarding the GC content, which optimal spans between 30 and 70%, post-optimization analysis revealed an average of 53.90%. The introduction of BamHI and XhoI sites at the respective ends of the codons enabled efficient integration into the MCS domain, with attention to specific restriction sites illustrated in **Supplementary Figure 12**.

### The simulation polymerase chain reaction and agarose gel electrophoresis

**Figure 8:**
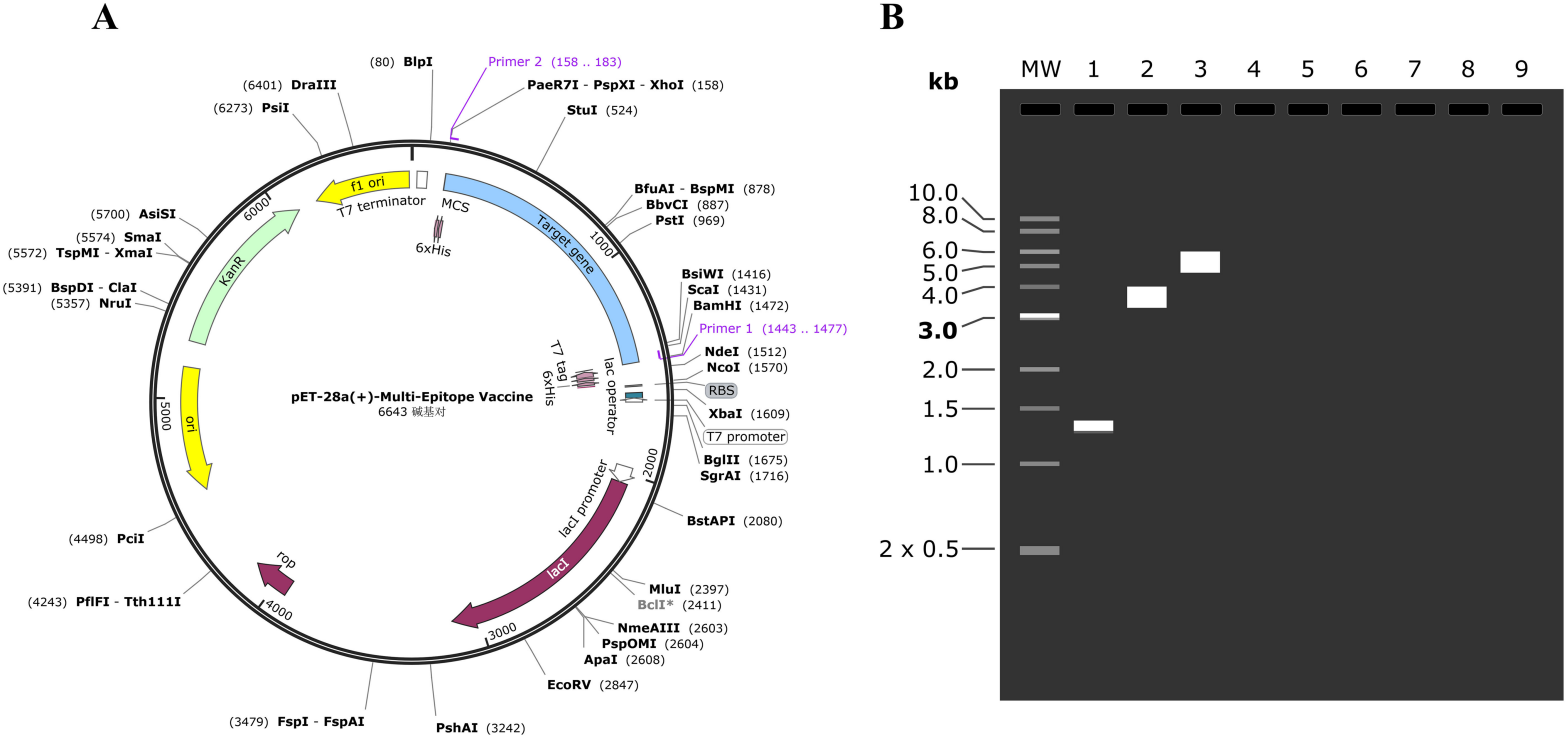
**(A)** The MEV (blue) was inserted into the pET28a (+) vector (black) in silico cloning. **(B)** The mock agarose gel electrophoresis experiments. As illustrated in the figure, “1” stands for the MEV, “2” stands for pET-28a (+), and “3” stands for recombinant plasmid.

The simulation PCR and agarose gel electrophoresis were conducted using primers designed according to specific criteria: the forward primer with a melting temperature (Tm) of 61 and a GC content of 69%, and the reverse primer with a Tm of 60 and 49% GC content. The target gene was amplified using SnapGene software, and agarose electrophoresis was simulated with a 1% concentration of TBE buffer for optimal buffering. The resulting DNA quantities were consistent with previous estimations. The sequence lengths were determined to be 1320 bp for the target gene, 5639 bp for pET-28a (+), and 6643 bp for the recombinant plasmid, as illustrated in **Figure 8**.

### Detecting the immunogenicity of MEV

**Figure 9:**
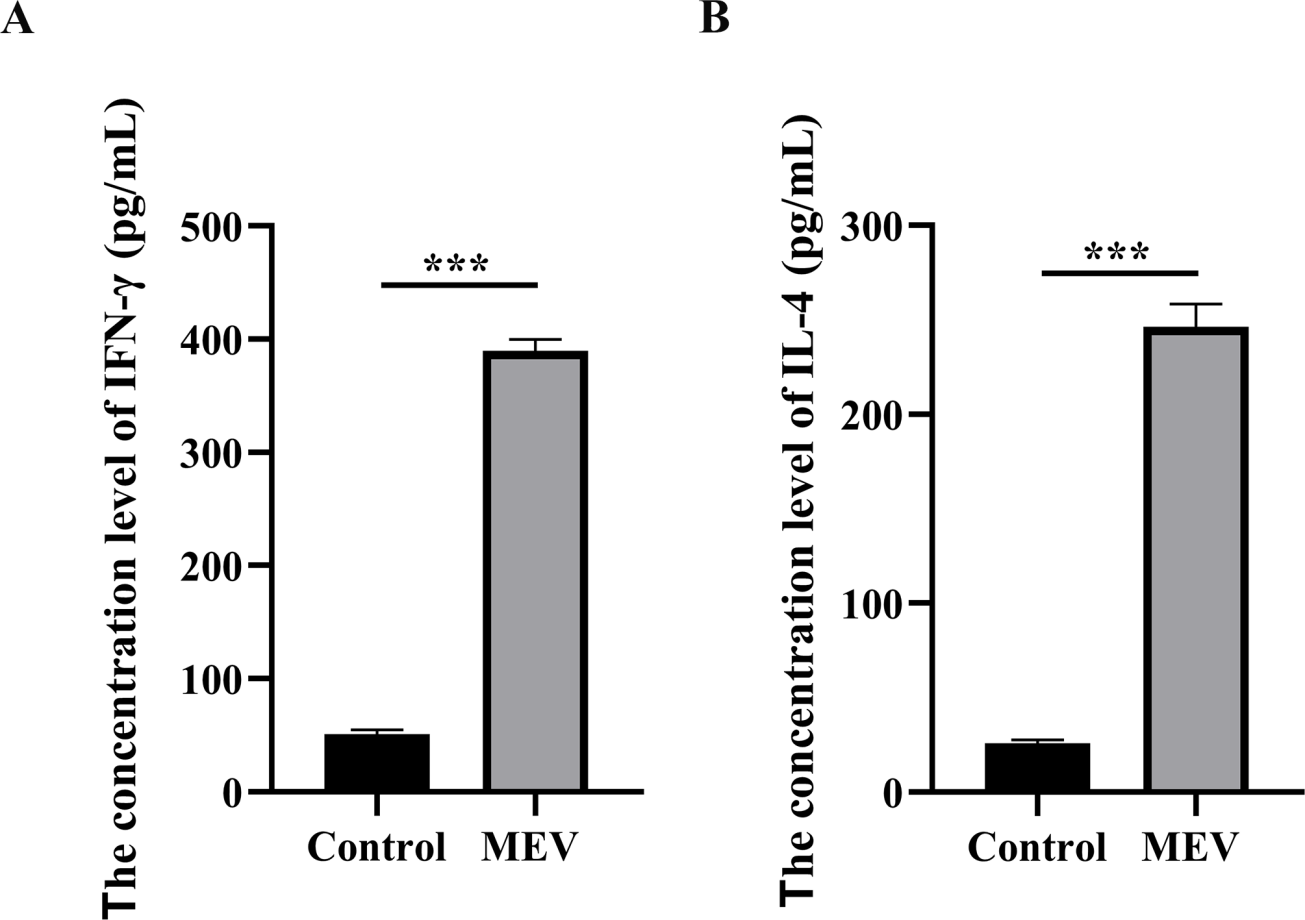
The results of *in vitro* experiment. **(A)** This diagram is the statistical analysis of IFN-γ concentration level between Control and MEV group, and the statistical method is two independent samples t-test (***P < 0.01). **(B)** This diagram is the statistical analysis of IL-4 concentration level between Control and MEV group, and the statistical method is two independent samples t-test (***P < 0.01).

We detected the secretion of IFN-γ and IL-4 following stimulation of PBMCs with MEV and PBS. The average IFN-γ secretion in the Control group was 50.86 pg/mL, while in the MEV group it was significantly elevated at 389.70 pg/mL. Similarly, the average IL-4 secretion in the Control group was 25.96 pg/mL, compared to 246.4 pg/mL in the MEV group. A two independent samples t-test was employed to assess the differences in secretion levels of IFN-γ and IL-4 between the Control and MEV groups. The IFN-γ secretion in the MEV group was significantly higher than that in the Control group (*P* < 0.001) (**Figure 9A**). Likewise, the IL-4 secretion in the MEV group was significantly greater than that in the Control group (*P* < 0.001) (**Figure 9B**).

### Detecting the antigenicity of MEV

**Figure 10:**
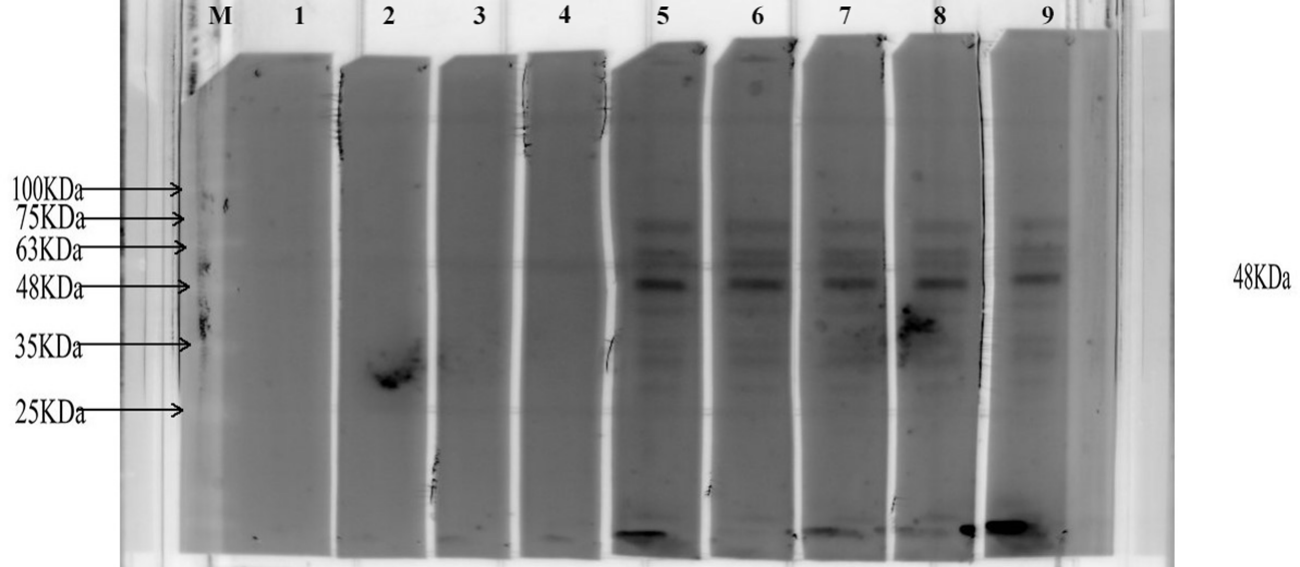
This diagram illustrates the representative Western Blot bands. The letter ‘M’ indicates the position of the marker. Bands 1-4 are derived from healthy individuals, while bands 5-9 are derived from patients with AE. The black band represents the imprint of the MEV protein.

The specific antibody against MEV in the serum of infected patients was assessed using Western Blot analysis. Consequently, when MEV protein was incubated with patient serum, a target band of approximately 48 kDa was produced. The results are presented in **Figure 10**.

### Detecting the immune response of mouse T-cell

**Figure 11:**
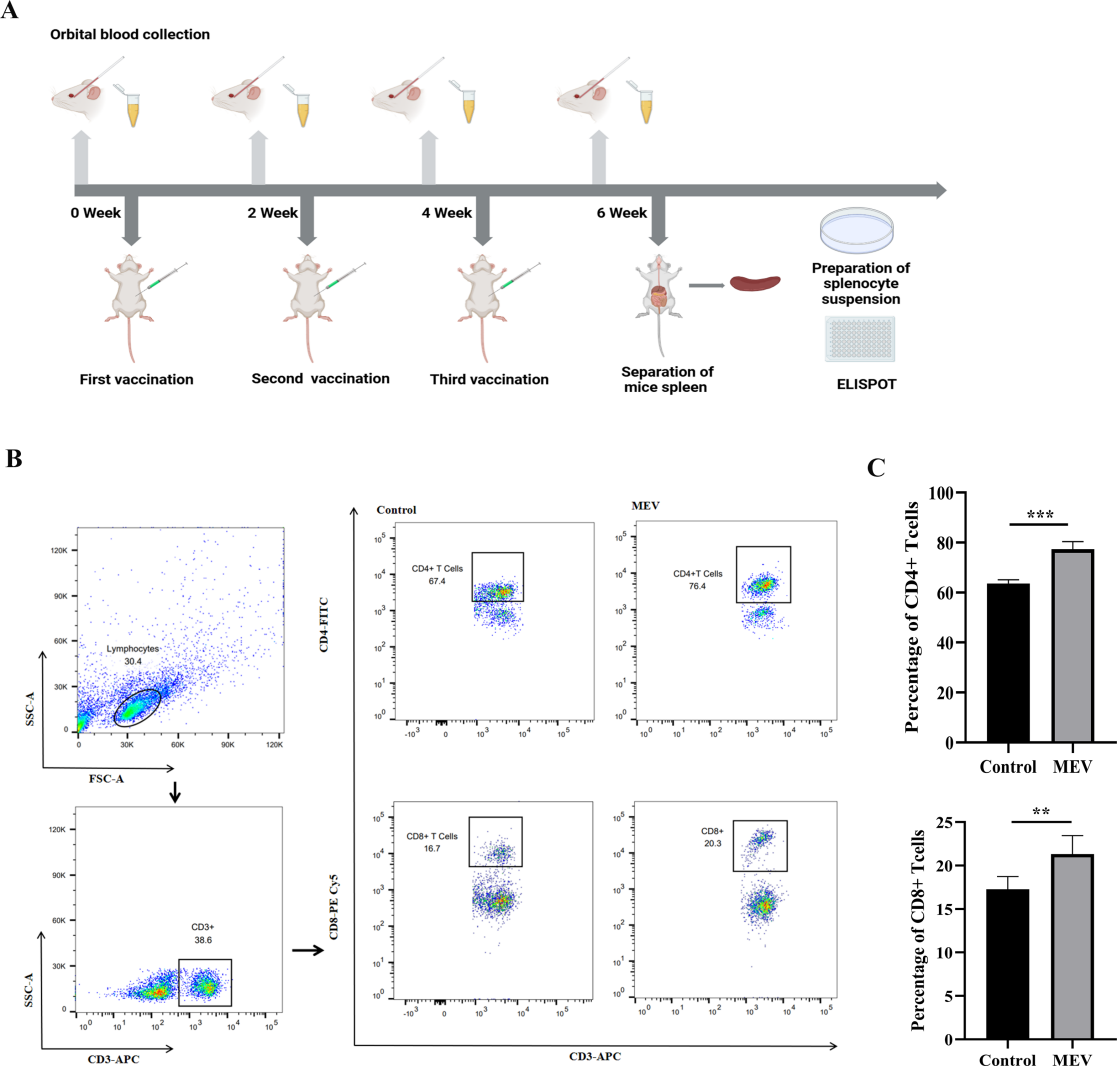
The results of the *in vivo* experiment. **(A)** The flow chart of mouse vaccination strategy, representing the steps of mouse vaccination experiment. **(B)** This is the representative FCM strategy diagram of CD4+ T-cells and CD8+ T-cells. **(C)** This diagram is the statistical analysis of CD4+ T cell and CD8+ T-cells percentages between Control and MEV group, and the statistical method is two independent samples t-test (*** *P* < 0.001, **P < 0.01).

**Figure 12:**
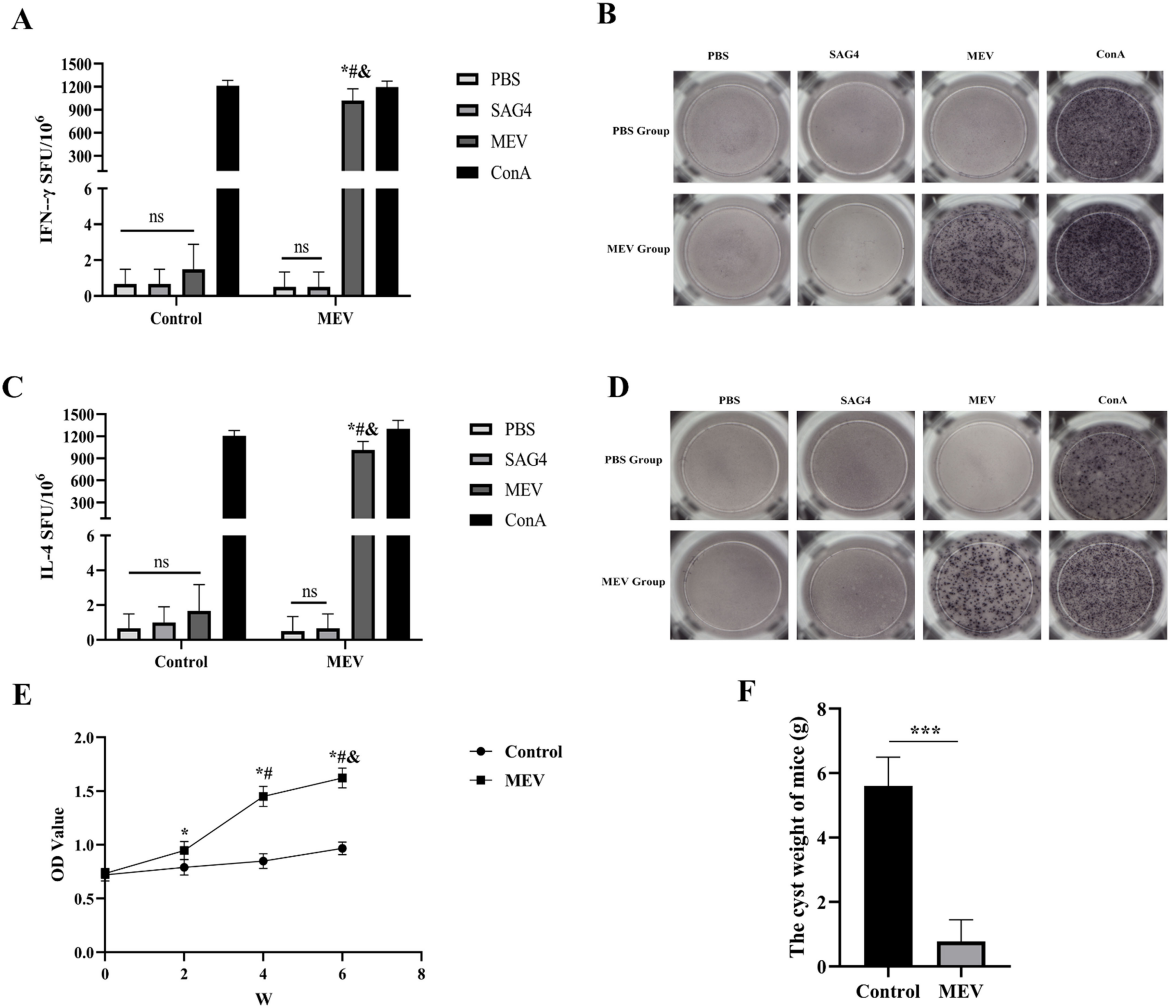
The results of the *in vivo* experiment. **(A)** The number of T-cells that can produce IFN-γ in splenocytes specimens after antigen stimulation. **(B)** The representative ELISPOT spot diagram of IFN-γ. **(C)** The number of T-cells that can produce IL-4 in splenocytes specimens after antigen stimulation. **(D)** The representative ELISPOT spot diagram of IL-4. One-way ANOVA was used for the Statistical Analysis. The * represents that compared with the Control group, *P* < 0.01. The # represents that in MEV group, compared with the PBS group, *P*<0.01. The & represents that in MEV group, compared with the SAG4 group, *P* < 0.01. ns *P* > 0.05. **(E)** Dynamic detection of specific antibody. The * represents that compared with the Control group, *P* < 0.01. The # represents that in MEV group, compared with the 2nd week, *P* < 0.01. The & represents that in MEV group, compared with the 4nd week, *P* < 0.01. **(F)** The weight of cysts. The difference in the weight of cysts between Control and MEV group was statistically significant. (***P < 0.001).

The flowchart illustrating the mouse vaccination strategy is presented in **Figure 11A**. FCM was employed to assess the levels of specific CD4+ T-cells and CD8+ T-cells (**Figure 11B**). The results showed that the levels of specific CD4+ T-cells and CD8+ T-cells in MEV group were significantly higher than those in Control group (*P* < 0.001, *P* < 0.01) (**Figure 11C**). The study evaluated the ability of the vaccine to induce a CD4+ T cell immune response by detecting the release of IFN-γ and IL-4 from mouse splenocytes through the ELISPOT assay. The results (**Figures 12A, C**) indicated that the MEV group produced significantly higher levels of IFN-γ and IL-4 compared to the Control group, with the differences being statistically significant (*P* < 0.05 for both), while significant differences were observed between the MEV group and both the PBS and SAG4 groups (*P* < 0.001 for both). The number of IFN-γ and IL-4 producing cells, visualized as spots, further illustrates the differences in results (**Figures 12B, D**). Our study demonstrates that MEV can effectively elicit a robust cellular immune response in an animal model.

### Detecting the immune response of mouse B-cell

To assess the antigenicity of MEV, mice were immunized with MEV protein, and serum was harvested to measure the levels of specific antibodies. As shown in **Figure 12E**, compared to the Control group, the levels of specific antibodies were significantly elevated two weeks after the first immunization (the 2nd week) in the MEV groups (*P* < 0.01), reaching their peak two weeks after the third immunization (the 6th week). Furthermore, the serum-specific antibody levels in the MEV groups at the 4 week were significantly elevated compared to baseline levels prior to MEV immunization (0 week, *P* < 0.01) and also when compared to levels measured 2 weeks after the first immunization (the second week, *P* < 0.01). These results indicated that MEV can enhance serum-specific antibody levels and activate humoral immunity in the animal model.

### Evaluation of potential protective effects of designed MEV

To verify the potential protective effects of the vaccine developed in this study, we established an animal infection model using mice challenged with *E. multilocularis* protoscolices. The results indicated a significant difference in cyst weight between the Control group and the MEV group (*P* < 0.001), as illustrated in **Figure 12F**. The average cyst weight in the MEV group was lower than that in the Control group, suggesting that the vaccine demonstrated a robust protective effect and effectively prevented infection by *E. multilocularis* protoscolices.

## Discussion

The development of the EG95 vaccine for cystic echinococcosis represents a significant breakthrough in controlling the transmission of *Echinococcus granulosus*. Additionally, it has enhanced our understanding of the parasite’s biology and immune responses, providing valuable insights for the potential development of vaccines against AE (39). The design of novel MEV, which consist of multiple protein epitopes, using immunoinformatics analyses has become a promising method to stimulate protective immune responses (40). The study utilized a variety of evaluation analyses, such as computational simulations, to thoroughly refine and validate the vaccine design. Follow-up experiments conducted in both laboratory and animal models provided additional evidence of the strong antigenicity and immunogenicity of the MEV. While previous study of MEV using reverse vaccinology have designed a GILE for *E. multilocularis*, our research aims to create a more comprehensive vaccine by predicting a wider range of T-cell epitopes and incorporating both linear and conformational B-cell epitopes, addressing the limitations of previous approaches (41). Two proteins, EmTSP3 and EmTIP, have been identified for their specific immunogenic properties (11). TSP3, used by Dang Z et al. in protective effects experiments, has shown that EmTSP3 elicits a significantly higher serum IgG immune response and reduces cyst lesions by 62.1%, compared to 32.1% with EmTSP1 (10). EmTIP, a homolog of human T-cell immunomodulatory proteins, is essential in the early stages of the parasite’s lifecycle. It enhances cell-cell/ECM interactions and stimulates the release of IFN-γ from mouse CD4+ T-cells, leading to Th1 polarization and ultimately restricting parasite proliferation (11, 42). Therefore, we aim to design an innovative MEV using epitopes from EmTSP3 and EmTIP to foster protective immunity against AE.

The transmembrane domains prediction in EmTSP3 and EmTIP are key to eliciting strong immune reactions by facilitating effective interaction with antigen-presenting cells, effector T-cells, and B-cells. The existence of signal peptides might influence the accurate folding of proteins or lead to their misplacement in incorrect cellular organelles, which could reduce vaccine effectiveness (43). Recognizing this, the signal peptide sequences of EmTSP3 (1–21) and EmTIP (1–20) were removed to allow for the entire amino acid sequence to undergo further analysis. The type and distribution of the protein’s secondary structure, along with the stability and functionality of its tertiary structure, collectively influence the antigenicity of the epitope (44). EmTSP3 exhibits a high α-helix content, while EmTIP predominantly adopts a random coil structure, which contributes to stable exposure and flexible availability, respectively (45). These features aid in identifying potential antigenic regions, including conformational epitopes, which are crucial for selecting effective epitopes for MEV development (46). The formation of complexes between HLA alleles and T-cell epitopes has demonstrated favorable affinity, suggesting the potential for effective immune response activation (47). T cell and B-cell epitopes are predicted and epitopes with good antigenicity, non-toxicity, and non-allergenicity are selected. The final selected epitopes of EmTSP3 and EmTIP are aligned in tandem to stimulate a stronger immune response, including humoral immunity and cellular immunity, thus enhancing the effectiveness of the vaccine (48, 49). Our MEV formulation also includes adjuvants such as hBD3 and the PADRE sequence to bolster immune protection (50, 51). After assembling the vaccine sequence, it was subjected to comprehensive analysis to evaluate its physicochemical properties, predict its secondary structure, construct its tertiary structure, and assess the model quality. These analytical results establish a solid foundation for further research on MEV. The favorable physicochemical properties indicate optimal safety and efficacy. With 47.94% alpha-helix content in the secondary structure, this high proportion may enhance the presentation of antigenic epitopes in multi-epitope vaccines (52). The high-quality tertiary structure model generated by AlphaFold2 will be instrumental in molecular docking, molecular dynamics simulation and structure optimization (33). This results will inform the design of subsequent *in vivo* and *in vitro* experiments, including antibody binding, T cell activation, and animal studies, ultimately expediting the vaccine development process and enhancing the likelihood of success (53). TLR4, a transmembrane receptor protein that recognizes pathogen-associated molecular patterns (PAMPs) and damage-associated molecular patterns (DAMPs), was shown through molecular docking to have strong intermolecular forces with the MEV, suggesting a promising capacity for activating the immune system (54, 55). This finding illustrates that MEV can form stable interactions with immune receptors, facilitating its distribution throughout the host’s body. To further evaluate MEV’s potential immune responses, as well as to validate the stability of its interactions with immune receptors, both immunological simulations and molecular dynamics simulations were conducted (56). The simulations revealed that vaccination with three doses led to a gradual increase in both B-cell and T-cell populations, peaking after the final dose. Furthermore, the MEV resulted in higher levels of key cytokines like IFN-γ, TGF-β, IL-10, and IL-2, crucial for coordinating immune responses. IFN-γ plays a vital role in cellular immunity by clearing pathogens, promoting Th1 cell differentiation, inducing B-cell isotype switching, enhancing NK cell cytotoxic responses, and stimulating T-cell antigen presentation (57). The simulations also showed an elevation in IgG levels, important for early host defense against infections through ADCC. These results suggest that MEV not only boosts immune cell numbers but also raises cytokine levels, strengthening the overall immune response and providing early protection against infections through increased IgG antibodies (58). The molecular dynamics simulations of the MEV-TLR4 complex demonstrate remarkable conformational stability and strong interactions with TLR4. This is evidenced by stable RMSD values, minimal RMSF fluctuations, and consistent RoG throughout the simulation, highlighting the structural integrity and compactness essential for immunogenic effectiveness. The identification of a stable low-energy conformational state within the Free Energy Landscape, along with favorable binding free energy calculations, indicates a robust and lasting interaction between the vaccine and TLR4, which is crucial for eliciting an effective immune response (47). The expression of MEV has been significantly enhanced through computer simulation optimization. The optimized vaccine protein demonstrated a CAI value of 0.82 and an average GC content of 69%. A recombinant plasmid consisting of 6643 base pairs was successfully constructed, providing a solid theoretical foundation for further research. This study conducted both *in vitro* and *in vivo* experiments to verify the immunogenicity and antigenicity of MEV. In the *in vitro* experiments, cellular immune function was first assessed using ELISA, revealing that the MEV protein significantly stimulates specific Th1 and Th2 lymphocytes to produce IFN-γ and IL-4. Shi et al. indicated that IFN-γ is induced by antigens, promoting the differentiation and development of Th1 cells and providing defense against parasites (59). It can also enhance the phagocytic function of macrophages and inhibit the proliferation of AE in patients. Wang et al. reported that Th2 cytokines allow parasites to proliferate at low rates by producing high levels of IL-4 (60). This dual activation suggests that MEV can effectively prime the immune system to respond to various stages of parasitic infection (61). The experimental results from Western Blot demonstrated that MEV could bind to specific antibodies present in the serum of AE patients, indicating effective antigenicity. In the *in vivo* experiments, the percentages of CD4+ T-cells and CD8+ T-cells in the splenocytes of mice in the MEV group were significantly higher than those in the control group. Furthermore, ELISPOT assays demonstrated that mice immunized with MEV were able to activate both Th1 and Th2 cell-mediated specific immune responses. Additionally, following each vaccine injection, the mice produced specific anti-MEV antibodies, with antibody levels gradually increasing in correlation with the number of injections. These findings indicate that MEV can effectively activate both cellular and humoral immunity in mice. Finally, the animal infection model demonstrated the protective effects of the MEV, with a significant reduction in cyst weight in the MEV group compared to the Control group. This finding suggests that the vaccine not only elicits a strong immune response but also translates into tangible protective effects against *E. multilocularis* infection.

## Conclusion

Our study employed various immunoinformatics analyses to identify beneficial epitopes of the EmTSP3 and EmTIP proteins. Following comprehensive analyses of allergenicity, toxicity, antigenicity, and molecular binding characteristics, the selected epitopes were conjugated to adjuvants using suitable linkers, leading to the formulation of the MEV structure. The efficacy of the vaccine was subsequently evaluated through molecular docking and molecular dynamics simulations, and its interaction with TLR4 was assessed. Our findings provide compelling evidence that the MEV can effectively activate both cellular and humoral immune responses, resulting in significant protective effects against parasitic infections. Collectively, these results suggest that MEV has the potential to serve as a highly effective vaccine candidate for the prevention of *E. multilocularis* infection.

## Data Availability

The datasets presented in this study can be found in online repositories. The names of the repository/repositories and accession number(s) can be found in the article/**Supplementary Material**.
