# Supplementary material for: Design of a novel EmTSP-3 and EmTIP based multi-epitope vaccine against Echinococcus multilocularis infection
Source: Front Immunol. 2024 Sep 16;15:1425603. doi: 10.3389/fimmu.2024.1425603 (PMC11439721; doi:10.3389/fimmu.2024.1425603)
Supplement: Supplementary file 1 [file DataSheet1.docx]

Supplementary Information for:

**Design of a Novel EmTSP-3 and EmTIP Based Multi-Epitope Vaccine Against *Echinococcus multilocularis* Infection.**

Yichen Fan^1†^, Yueyue He^2†^, Yujiao Li^4^, Zhengwei Yin^3^, Juan Shi^3^, Tingting Tian^3^, Kaiyu Shang^3^, Huidong Shi^3^, Fengbo Zhang^1,3*^ and Hao Wen^1*^

1 State Key Laboratory of Pathogenesis, Prevention and Treatment of High Incidence Diseases in Central Asia, Clinical Medicine Institute, The First Affiliated Hospital of Xinjiang Medical University, Urumqi, Xinjiang, China.

2 Department of Immunology, School of Basic Medical Sciences, Xinjiang Medical University, Urumqi, China.

3 Department of Clinical Laboratory, The First Affiliated Hospital of Xinjiang Medical University, Urumqi, China.

4 Department of Blood Transfusion, The First Affiliated Hospital of Xinjiang Medical University, Urumqi, China.

†Yichen Fan and Yueyue He contributed equally to this work.

*Corresponding authors: Hao Wen and Fengbo Zhang.

E-mail: [Dr.wenhao@163.com](mailto:Dr.wenhao@163.com) (Hao Wen); zfb131@163.com (Fengbo Zhang);

# Table and Figure of contents

**Supplemental Table 1-1.** The amino acid sequence of EmTSP3 and EmTIP.

**Supplemental Table 1-2.** DNASTAR and SOMPA predicted the secondary structures of Protein EmTSP3.

**Supplemental Table 1-3.** DNASTAR and SOMPA predicted the secondary structures of Protein EmTIP.

**Supplemental Table 2-1.** The predicted CD8+ T-cell epitopes of Protein EmTSP3 by IEDB and NetCTLpan.

**Supplemental Table 2-2.** The predicted CD8+ T-cell epitopes of Protein EmTIP by IEDB and NetCTLpan.

**Supplemental Table 3-1.** The predicted CD4+ T-cell epitopes of Protein EmTSP3 by IEDB and NetMHCIIpan.

**Supplemental Table 3-2.** The predicted CD4+ T-cell epitopes of Protein EmTIP by IEDB and NetMHCIIpan.

**Supplemental Table 4-1.** The predicted B-cell epitopes of Protein EmTSP3 by DNASTAR and IEDB.

**Supplemental Table 4-2.** The predicted B-cell epitopes of Protein EmTIP by DNASTAR and IEDB.

**Supplemental Table 4-3.** The predicted B-cell epitopes of Protein EmTSP3 by IEDB.

**Supplemental Table 4-4.** The predicted B-cell epitopes of Protein EmTIP by IEDB.

**Supplemental Figure 1.** The Protein EmTSP3 have 2 transmembrane domains and the Protein EmTIP had 1 transmembrane domain.

**Supplemental Figure 2.** Signal peptide of Protein EmTSP3 and EmTIP.

**Supplemental Figure 3.** NetPhos 3.1 analysis showed Protein EmTSP3 has 6 serine, 3 threonine, and 9 tyrosine phosphorylation sites, mainly targeted by PKC, PKA, and CKI. Protein EmTIP has 39 serine, 21 threonine, and 5 tyrosine phosphorylation sites, mainly targeted by PKC, PKA, DNA-PK, and cDC2.

**Supplemental Figure 4.** The secondary structure prediction of Protein EmTSP3 and EmTIP was conducted using DNASTAR. The Gramier-Robson method, represented by lines A, B, I, and C, utilized colors to indicate different structural elements: α-helix (red), β-fold (green), β-turn (blue), and random coil (yellow). Additionally, the Chou-Fasman method employed lines A, E, and T to depict the same structural elements without including a random coil structure.

**Supplemental Figure 5-1.** Secondary structure of Protein EmTSP3 by SOPMA. The result of server
SOPMA shows that the blue h is Alpha helix, the red e is Extended strand, the green t is Beta turn, the yellow c is Random coil. Protein EmTSP3 consisted of 60.81% Alpha helix, 9.46% Extended strand, 2.7% Beta turn, and 27.03% Random coil structures.

**Supplemental Figure 5-2.** Secondary structure of Protein EmTIP by SOPMA. The result of server SOPMA shows that the blue h is Alpha helix, the red e is Extended strand, the green t is Beta turn, the yellow c is Random coil. Protein EmTIP consisted of 15.71% Alpha helix, 30.07% Extended strand, 3.21% Beta turn, and 51.01% Random coil structures

**Supplemental Figure 6.** (A) Tertiary structures of Protein EmTSP3. (B) Tertiary structures of Protein EmTIP. Next to each structure is a Ramachandran plot indicating allowed and disallowed rotation angles of peptide bonds. Inside the dark green circle represents completely allowed areas, inside the light green circle represents allowed areas, and outside the green circle is not allowed. A good model has over 90% of scatter within these circles.

**Supplemental Figures 7.** B-cell epitopes of Protein EmTSP3 and EmTIP by DNASTAR. The yellow area represents Surface accessibility, the blue area represents Flexibility, the pink area represents Antigenicity and the green area represents Hydrophobicity. We need to obtain the areas above the threshold of the purple area, and the rectangles on the other three lines.

**Supplemental Figures 8-1.** B-cell epitopes of Protein EmTSP3 by IEDB. The A1 is Surface accessibility, the B1 is Flexibility, the C1 is Antigenicity, the D1 is Hydrophilicity. The yellow area represents primary result.

**Supplemental Figures 8-2.** B-cell epitopes of Protein EmTIP by IEDB. The A2 is Surface accessibility, the B2 is Flexibility, the C2 is Antigenicity, the D2 is Hydrophilicity. The yellow area represents primary result.

**Supplemental Figure 9.** Results of the HLA-peptide complexes. (A) HLA-A*02:01 and CTL epitopes docking result. (B) HLA-DRB1*01:01 and HTL epitopes docking result.

**Supplemental Figure 10.** The top chart illustrates the length of the query sequence, approximately 430 amino acids or nucleotides. A matching region at the beginning (positions 1-80) is highlighted by a pink bar. This indicates high similarity to the known Beta-defensin 3 protein at the beginning, covering only about 10% of the entire sequence.

**Supplemental Figure 11.** Protein conformation at the low point of the Free Energy Landscape.

**Supplemental Figure 12.** (A) Adaptiveness of optimized codon. (B) The GC content. (C) The MEV (1320 bp) after amplified.

| **Supplemental Table 1-1.** The amino acid sequence of EmTSP3 and EmTIP. | |
| --- | --- |
| Protein | Amino acid sequence |
| TSP3 | MLKTFAVIVAILLVAEIVCGIVLLVYRHEFVGLVGKEMQREIKDLTAHGRNASDPLLKSIYKLQEELECCGGVGPTDWSKPYPASCCKSGKENCTQPYQQGCAVAMYEQIKDSSLAFGLIILIVCLIQIGAVICACCLAKKVNEYEKV |
| TIP | MQAYAYILTVWFLGVQCVAITGPGGSSFSLVASWSDADLAAFADVNTDRRTDAIVLSSSESALYALLAPNAKQTDGKPERKKLFSLTHSLPLRSIAVADFDGDSVADFLLVFRDTSKYSVNVWYGGKNVTRSVGSFATQPLVCDVNADMIADVYGEMENHRVVALGEPNAFSLQNFTHSATSLSELSSAGFVSLAFNPNPSLVTLAVDTIEVFNDLTPKGDGSPSSYPLPIELKGANRPIGKLVFGDFDMSGRIQLLIAGCSDNTCRHSYIFMHSLTGNAVWEAIAVEWNPPEMEGSCSLAPASVDQFSSAAIIGLSLGDADLDGYPDLAVGLKCTRSSGLRPIILPAILRNLAGAGRKVRFQAYLLPGVESQETLKQIAFYDYNEDGILDLYMSYEGRGGVSTSLYMQKLTKEAYFLKVMLTTGRCGSPSQCPDGVLPYGLPGYGFRASYETQGADGGRIRSSAAFVTSSCCGALQLPFTTFGFGDFATYIENVPVSVPAPTQQARKHKLTFIVPNAQVVVVPYPPDNPSNWQAKLFLQPLYDMKVIYVAITLLVTCIVLLVVVGILQYLEVRSDQKERMQESQRFHFDAM |

| **Supplemental Table 1-2.** DNASTAR and SOMPA predicted the secondary structures of Protein EmTSP3. | | | | |
| --- | --- | --- | --- | --- |
| Methods | α-Helix | β-Strand | β-Turn | Random Coil |
| DNASTAR |  |  |  |  |
| Garnier-Robson | 1-27 33-47 82-101 106-107 111-127 | 81 102-105 | 29 31 48-51 55-57 60-74 76-80 108-110 | 28 30 32 52-54 58-59 75 |
| Chou-Fasman | 5-10 12-24 39-47 81-90 120-127 | 1-4 7-14 35-41 95-119 | 25-28 30-33 48-51 54-57 59-62 66-73 75-78 91-94 |  |
| SOMPA | 1-2 5-26 25-47 82-92 98-121 126-127 | 3-4 94-97 |  | 27-34 48-82 122-125 |

| **Supplemental Table 1-3.** DNASTAR and SOMPA predicted the secondary structures of Protein EmTIP. | | | | |
| --- | --- | --- | --- | --- |
| Methods | α-Helix | β-Strand | β-Turn | Random Coil |
| DNASTAR |  |  |  |  |
| Garnier-Robson | 13-27 29-30 39-54 57-64 83-88 91-94 125-144 149-154 163-170 214-215 230-234 260-267 277-282 285-290 351-358 367-370 387-400 482-488 521-525 553-572 | 7-12 31-37 65-67 73-80 99-104 109-124 171-175 182-194 207-213 218-227 235-239 249-254 291-308 311-317 321-332 338-346 359-363 371-377 383-386 401-404 412-414 416-423 425-233 440-450 455-463 470-481 489-496 498-505 515-520 526-552 | 95-98 198-201 240-248 364-366 405-408 434-437 451-454 | 69-71 157-162 176-181 255-259 269-272 347-350 466-469 |
| Chou-Fasman | 18-25 42-47 57-65 72-80 124-131 136-146 162-166 172-176 209-214 260-270 298-301 325-334 339-344 350-354 369-374 387-404 443-448 485-488 513-519 558-572 | 10-13 31-37 66-68 85-92 98-102 107-111 116-123 172-175 181-195 220-226 233-240 250-255 290-296 322-333 345-347 355-361 445-449 457-463 467-477 489-494 499-502 524-553 | 2-9 14-17 26-29 38-41 48-51 53-56 81-84 94-97 103-106 112-115 132-135 147-150 168-171 177-180 197-200 203-206 215-218 229-232 241-244 246-249 256-259 271-274 276-279 284-287 302-309 318-321 335-338 365-368 378-381 383-386 405-416 418-425 428-431 435-438 450-453 481-484 495-498 503-506 509-512 520-523 554-557 |  |
| SOMPA | 395-396 442-445 488-491 520-544 547-552 555-572 | 9-11 19-22 32-37 42-46 72-78 87-91 101-104 121-124 132-134 145-146 166-167 171-172 181-184 190-194 212-213 222-227 234-239 249-251 280-283 294-299 308-313 322-326 342-345 358-363 371-375 384-388 397-403 429-434 441-442 460-464 475-477 492-494 500-503 513-519 545-546 |  | 1-8 12-18 24-31 38-42 47-71 79-86 92-100 105-120 125-131 135-144 147-165 168-170 174-180 185-189 195-211 214-221 228-233 240-248 252-279 284-293 300-307 314-322 327-341 346-357 364-370 376-383 389-394 404-423 435-440 446-459 465-470 478-497 495-499 504-512 520-526 553-554 |

| **Supplemental Table 2-1.** The predicted CD8+ T-cell epitopes of Protein EmTSP3 by IEDB and NetCTLpan. | | | | | | | |
| --- | --- | --- | --- | --- | --- | --- | --- |
| Methods | Allele | Serials | Sequence | rank | Antigenicity | Allergenicity | Toxicity |
| IEDB | HLA-A*11:01 | 31-41 | ASDPLLKSIYK | 0.2 | 0.52 | Non-Allergen | Non-Toxin |
|  |  | 30-41 | NASDPLLKSIYK | 0.26 | 0.4 | Non-Allergen | Non-Toxin |
|  |  | 110-119 | AVICACCLAK | 0.27 | 0.12 | Non-Allergen | Non-Toxin |
|  |  | 29-41 | RNASDPLLKSIYK | 0.3 | 0.12 | Non-Allergen | Non-Toxin |
|  |  | 28-41 | GRNASDPLLKSIYK | 0.31 | 0.39 | Non-Allergen | Non-Toxin |
|  |  | 111-119 | VICACCLAK | 0.45 | -0.06 | Non-Allergen | Non-Toxin |
|  |  | 51-59 | GVGPTDWSK | 1.08 | 0.57 | Non-Allergen | Non-Toxin |
|  |  | 111-120 | VICACCLAKK | 1.11 | -0.55 | Non-Allergen | Non-Toxin |
|  | HLA-A*03:01 | 110-119 | AVICACCLAK | 0.36 | 0.12 | Non-Allergen | Non-Toxin |
|  |  | 3-15 | LVYRHEFVGLVGK | 0.4 | 0.89 | Non-Allergen | Non-Toxin |
|  |  | 111-119 | VICACCLAK | 0.45 | -0.06 | Non-Allergen | Non-Toxin |
|  |  | 2-15 | LLVYRHEFVGLVGK | 0.54 | 1.06 | Non-Allergen | Non-Toxin |
|  |  | 111-120 | VICACCLAKK | 0.67 | -0.55 | Non-Allergen | Non-Toxin |
|  |  | 24-37 | LTAHGRNASDPLLK | 0.9 | 1.01 | Non-Allergen | Non-Toxin |
|  |  | 3-16 | LVYRHEFVGLVGKE | 0.98 | 0.83 | Non-Allergen | Non-Toxin |
|  | HLA-A*02:01 | 93-102 | SLAFGLIILI | 0.9 | 0.55 | Non-Allergen | Non-Toxin |
|  |  | 100-108 | ILIVCLIQI | 0.77 | 0.82 | Probable-Allergen | Non-Toxin |
|  |  | 93-101 | SLAFGLIIL | 0.9 | 0.55 | Non-Allergen | Non-Toxin |
|  |  | 92-102 | SSLAFGLIILI | 1.05 | 0.94 | Probable-Allergen | Non-Toxin |
| NetCTLpan | HLA-A*11:01 | 30-40 | ASDPLLKSIYK | 0.3 | 0.52 | Non-Allergen | Non-Toxin |
|  |  | 109-119 | AVICACCLAKK | 1.5 | -0.31 | Non-Allergen | Non-Toxin |
|  |  | 115-125 | CLAKKVNEYEK | 1.5 | -0.07 | Non-Allergen | Non-Toxin |
|  |  | 56-66 | WSKPYPASCCK | 2 | 0.8 | Non-Allergen | Non-Toxin |
|  | HLA-A*03:01 | 115-125 | CLAKKVNEYEK | 1 | -0.07 | Non-Allergen | Non-Toxin |
|  |  | 30-40 | ASDPLLKSIYK | 1.5 | 0.52 | Non-Allergen | Non-Toxin |
|  |  | 56-66 | WSKPYPASCCK | 3 | 0.8 | Non-Allergen | Non-Toxin |
|  | HLA-A*02:01 | 83-97 | VAMYEQIKDSSLAFG | 0.8 | 0.71 | Non-Allergen | Non-Toxin |
|  |  | 92-102 | SLAFGLIILIV | 0.8 | 0.63 | Non-Allergen | Non-Toxin |
|  |  | 92-101 | SLAFGLIILI | 0.8 | 0.69 | Non-Allergen | Non-Toxin |

| **Supplemental Table 2-2.** The predicted CD8+ T-cell epitopes of Protein EmTIP by IEDB and NetCTLpan. | | | | | | | |
| --- | --- | --- | --- | --- | --- | --- | --- |
| Methods | Allele | Serials | Sequence | rank | Antigenicity | Allergenicity | Toxicity |
| IEDB | HLA-A*11:01 | 84-97 | SVADFLLVFRDTSK | 0.05 | -0.47 | Non-Allergen | Non-Toxin |
|  |  | 205-214 | SSYPLPIELK | 0.11 | 0.98 | Non-Allergen | Non-Toxin |
|  |  | 203-214 | SPSSYPLPIELK | 0.16 | 0.96 | Non-Allergen | Non-Toxin |
|  |  | 381-390 | GVSTSLYMQK | 0.16 | 0.69 | Non-Allergen | Non-Toxin |
|  |  | 201-214 | DGSPSSYPLPIELK | 0.17 | 1.21 | Non-Allergen | Non-Toxin |
|  |  | 202-214 | GSPSSYPLPIELK | 0.17 | 1.11 | Non-Allergen | Non-Toxin |
|  |  | 84-93 | SVADFLLVFR | 0.26 | -0.53 | Non-Allergen | Non-Toxin |
|  |  | 384-393 | TSLYMQKLTK | 0.24 | 0.73 | Non-Allergen | Non-Toxin |
|  |  | 382-392 | VSTSLYMQKLTK | 0.27 | 0.8 | Non-Allergen | Non-Toxin |
|  |  | 99-107 | SVNVWYGGK | 0.27 | 0.28 | Non-Allergen | Non-Toxin |
|  |  | 382-390 | VSTSLYMQK | 0.29 | 0.98 | Non-Allergen | Non-Toxin |
|  | HLA-A*03:01 | 385-393 | SLYMQKLTK | 0.11 | 0.73 | Non-Allergen | Non-Toxin |
|  |  | 515-526 | AKLFLQPLYDMK | 0.16 | 1.14 | Non-Allergen | Non-Toxin |
|  |  | 514-526 | QAKLFLQPLYDMK | 0.19 | 1.15 | Probable-Allergen | Non-Toxin |
|  |  | 513-526 | WQAKLFLQPLYDMK | 0.2 | 1.7 | Probable-Allergen | Non-Toxin |
|  |  | 516-526 | KLFLQPLYDMK | 0.25 | 1.2 | Probable-Allergen | Non-Toxin |
|  |  | 40-51 | SALYALLAPNAK | 0.28 | 0.64 | Non-Allergen | Non-Toxin |
|  |  | 205-214 | SSYPLPIELK | 0.29 | 0.98 | Non-Allergen | Non-Toxin |
|  | HLA-A*02:01 | 518-530 | FLQPLYDMKVIYV | 0.24 | 0.93 | Probable-Allergen | Non-Toxin |
| NetCTLpan | HLA-A*11:01 | 382-392 | STSLYMQKLTK | 0.2 | 0.67 | Non-Allergen | Non-Toxin |
|  |  | 41-51 | ALYALLAPNAK | 0.3 | 0.77 | Non-Allergen | Non-Toxin |
|  |  | 460-470 | TTFGFGDFATY | 0.3 | -0.06 | Non-Allergen | Non-Toxin |
|  |  | 188-198 | TIEVFNDLTPK | 0.8 | 0.11 | Non-Allergen | Non-Toxin |
|  | HLA-A*03:01 | 40-51 | SALYALLAPNAK | 0.05 | 0.64 | Probable-Allergen | Non-Toxin |
|  |  | 382-392 | STSLYMQKLTK | 0.8 | 0.67 | Non-Allergen | Non-Toxin |
|  |  | 515-525 | KLFLQPLYDMK | 0.8 | 1.2 | Probable-Allergen | Non-Toxin |
|  |  | 539-549 | VLLVVVGILQY | 0.8 | 0.57 | Non-Allergen | Non-Toxin |
|  | HLA-A*02:01 | 205-214 | KLFSLTHSLPL | 0.2 | 0.13 | Non-Allergen | Non-Toxin |
|  |  | 161-171 | SLSELSSAGFV | 0.8 | 0.48 | Probable-Allergen | Non-Toxin |

| **Supplemental Table 3-1.** The predicted CD4+ T-cell epitopes of Protein EmTSP3 by IEDB and NetMHCIIpan. | | | | | | | |
| --- | --- | --- | --- | --- | --- | --- | --- |
| Methods | Allele | Serials | Sequence | rank | Antigenicity | Allergenicity | Toxicity |
| IEDB | HLA-DRB1*07:01 | 6-20 | RHEFVGLVGKEMQRE | 2.9 | 1.18 | Non-Allergen | Non-Toxin |
|  |  | 5-19 | YRHEFVGLVGKEMQR | 3.7 | 1.16 | Non-Allergen | Non-Toxin |
|  |  | 83-96 | VAMYEQIKDSSLAF | 3.8 | 0.71 | Non-Allergen | Non-Toxin |
|  |  | 82-96 | AVAMYEQIKDSSLAF | 4.9 | 0.65 | Non-Allergen | Non-Toxin |
|  |  | 81-95 | CAVAMYEQIKDSSLA | 5.3 | 0.71 | Non-Allergen | Non-Toxin |
|  |  | 4-18 | VYRHEFVGLVGKEMQ | 6.2 | 1.04 | Non-Allergen | Non-Toxin |
|  |  | 7-21 | HEFVGLVGKEMQREI | 8.9 | 0.91 | Non-Allergen | Non-Toxin |
|  |  | 74-88 | TQPYQQGCAVAMYEQ | 9.7 | 0.56 | Non-Allergen | Non-Toxin |
|  |  | 84-98 | AMYEQIKDSSLAFGL | 11 | 0.61 | Non-Allergen | Non-Toxin |
|  | HLA-DRB1*03:01 | 14-28 | GKEMQREIKDLTAHG | 7.6 | 1.3 | Non-Allergen | Non-Toxin |
|  |  | 33-47 | DPLLKSIYKLQEELE | 9 | 0.21 | Non-Allergen | Non-Toxin |
|  |  | 13-27 | VGKEMQREIKDLTAH | 0.3 | 0.62 | Non-Allergen | Non-Toxin |
|  |  | 18-32 | QREIKDLTAHGRNAS | 11 | 1.39 | Non-Allergen | Non-Toxin |
|  |  | 21-35 | IKDLTAHGRNASDPL | 12 | 1.25 | Non-Allergen | Non-Toxin |
|  |  | 32-46 | SDPLLKSIYKLQEEL | 12 | 0.3 | Non-Allergen | Non-Toxin |
|  | HLA-DRB1*15:01 | 18-32 | QREIKDLTAHGRNAS | 5.3 | 1.39 | Non-Allergen | Non-Toxin |
|  |  | 17-31 | MQREIKDLTAHGRNA | 6.3 | 1.25 | Non-Allergen | Non-Toxin |
|  |  | 6-20 | RHEFVGLVGKEMQRE | 7.6 | 1.18 | Non-Allergen | Non-Toxin |
|  |  | 16-30 | EMQREIKDLTAHGRN | 10 | 1.24 | Probable-Allergen | Non-Toxin |
|  |  | 5-19 | YRHEFVGLVGKEMQR | 11 | 1.16 | Non-Allergen | Non-Toxin |
| NetMHCIIpan | HLA-DRB1*07:01 | 6-20 | RHEFVGLVGKEMQRE | 1.88 | 1.18 | Non-Allergen | Non-Toxin |
|  |  | 83-96 | VAMYEQIKDSSLAF | 2.38 | 0.71 | Non-Allergen | Non-Toxin |
|  |  | 5-19 | YRHEFVGLVGKEMQR | 2.48 | 1.16 | Non-Allergen | Non-Toxin |
|  |  | 82-96 | AVAMYEQIKDSSLAF | 3.2 | 0.65 | Non-Allergen | Non-Toxin |
|  |  | 81-95 | CAVAMYEQIKDSSLA | 3.84 | 0.71 | Non-Allergen | Non-Toxin |
|  |  | 4-18 | VYRHEFVGLVGKEMQ | 4.49 | 1.04 | Non-Allergen | Non-Toxin |
|  |  | 7-21 | HEFVGLVGKEMQREI | 6.5 | 0.91 | Non-Allergen | Non-Toxin |
|  | HLA-DRB1*03:01 | 14-28 | GKEMQREIKDLTAHG | 5.59 | 1.3 | Non-Allergen | Non-Toxin |
|  | HLA-DRB1*15:01 | 18-32 | QREIKDLTAHGRNAS | 3.59 | 1.39 | Non-Allergen | Non-Toxin |
|  |  | 17-31 | MQREIKDLTAHGRNA | 4.22 | 1.25 | Non-Allergen | Non-Toxin |

| **Supplemental Table 3-2.** The predicted CD4+ T-cell epitopes of Protein EmTIP by IEDB and NetMHCIIpan. | | | | | | | |
| --- | --- | --- | --- | --- | --- | --- | --- |
| Methods | Allele | Serials | Sequence | rank | Antigenicity | Allergenicity | Toxicity |
| IEDB | HLA-DRB1*07:01 | 60-74 | RKKLFSLTHSLPLRS | 0.03 | 0.3 | Non-Allergen | Non-Toxin |
|  |  | 61-75 | KKLFSLTHSLPLRSI | 0.04 | -0.01 | Non-Allergen | Non-Toxin |
|  |  | 59-73 | ERKKLFSLTHSLPLR | 0.06 | 0.08 | Non-Allergen | Non-Toxin |
|  |  | 62-76 | KLFSLTHSLPLRSIA | 0.17 | 0.13 | Non-Allergen | Non-Toxin |
|  |  | 58-72 | PERKKLFSLTHSLPL | 0.28 | 0.04 | Non-Allergen | Non-Toxin |
|  | HLA-DRB1*03:01 | 549-563 | QYLEVRSDQKERMQE | 0.07 | 1.5 | Non-Allergen | Non-Toxin |
|  |  | 550-564 | YLEVRSDQKERMQES | 0.07 | 1.59 | Non-Allergen | Non-Toxin |
|  |  | 551-565 | LEVRSDQKERMQESQ | 0.12 | 1.72 | Probable-Allergen | Non-Toxin |
|  |  | 548-562 | LQYLEVRSDQKERMQ | 0.22 | 1.13 | Probable-Allergen | Non-Toxin |
|  |  | 21-35 | AFADVNTDRRTDAIV | 0.93 | 0.72 | Non-Allergen | Non-Toxin |
|  | HLA-DRB1*15:01 | 367-381 | DGILDLYMSYEGRGG | 0.08 | 0.61 | Non-Allergen | Non-Toxin |
|  |  | 366-380 | EDGILDLYMSYEGRG | 0.21 | 0.51 | Non-Allergen | Non-Toxin |
|  |  | 300-314 | DADLDGYPDLAVGLK | 0.29 | 0.7 | Non-Allergen | Non-Toxin |
|  |  | 299-313 | GDADLDGYPDLAVGL | 0.65 | 0.76 | Probable-Allergen | Non-Toxin |
|  |  | 16-30 | DADLAAFADVNTDRR | 0.65 | 0.63 | Non-Allergen | Non-Toxin |
|  |  | 544-558 | VVGILQYLEVRSDQK | 0.66 | 0.9 | Probable-Allergen | Non-Toxin |
|  |  | 368-382 | GILDLYMSYEGRGGV | 0.81 | 0.27 | Non-Allergen | Non-Toxin |
|  |  | 365-379 | NEDGILDLYMSYEGR | 0.87 | 0.33 | Non-Allergen | Non-Toxin |
|  |  | 110-124 | TRSVGSFATQPLVCD | 0.88 | 0.38 | Non-Allergen | Non-Toxin |
|  |  | 281-295 | APASVDQFSSAAIIG | 0.97 | 0.29 | Non-Allergen | Non-Toxin |
| NetMHCIIpan | HLA-DRB1*07:01 | 60-74 | RKKLFSLTHSLPLRS | 0 | 0.3 | Non-Allergen | Non-Toxin |
|  |  | 61-75 | KKLFSLTHSLPLRSI | 0 | -0.01 | Non-Allergen | Non-Toxin |
|  |  | 59-73 | ERKKLFSLTHSLPLR | 0.02 | 0.08 | Non-Allergen | Non-Toxin |
|  |  | 62-76 | KLFSLTHSLPLRSIA | 0.08 | 0.13 | Non-Allergen | Non-Toxin |
|  |  | 58-72 | PERKKLFSLTHSLPL | 0.14 | 0.04 | Non-Allergen | Non-Toxin |
|  | HLA-DRB1*03:01 | 550-564 | YLEVRSDQKERMQES | 0.04 | 1.59 | Probable-Allergen | Non-Toxin |
|  |  | 549-563 | QYLEVRSDQKERMQE | 0.06 | 1.5 | Probable-Allergen | Non-Toxin |
|  |  | 551-564 | LEVRSDQKERMQES | 0.15 | 1.72 | Probable-Allergen | Non-Toxin |
|  | HLA-DRB1*15:01 | 367-381 | DGILDLYMSYEGRGG | 0.08 | 0.61 | Non-Allergen | Non-Toxin |
|  |  | 366-380 | EDGILDLYMSYEGRG | 0.16 | 0.51 | Non-Allergen | Non-Toxin |

| **Supplemental Table 4-1.** The predicted B-cell epitopes of Protein EmTSP3 by DNASTAR and IEDB. | | |
| --- | --- | --- |
| Software | Analysis Parameters | The areas of prediction of epitope |
| DNASTAR | Surface Accessibility | 16-32 41-44 55-61 72-77 87-90 120-127 |
|  | Flexibility | 17-37 42-45 51-61 65-83 88-93 119-124 |
|  | Antigenicity | 13-36 40-62 63-82 87-94 117-127 |
|  | Hydrophilicity | 15-34 40-47 53-83 87-91 119-127 |
| IEDB | Surface Accessibility | 15-22 27-32 55-61 64-88 72-77 |
|  | Flexibility | - |
|  | Antigenicity | 45-51 62-67 78-84 95-119 |
|  | Hydrophilicity | - |

| **Supplemental Table 4-2.** The predicted B-cell epitopes of Protein EmTIP by DNASTAR and IEDB. | | |
| --- | --- | --- |
| Software | Analysis Parameters | The areas of prediction of epitope |
| DNASTAR | Surface Accessibility | 25-31 50-62 94-98 196-207 269-274 338-341 350-356 362-366 373-378 389-394 429-434 481-496 505-514 553-567 |
|  | Flexibility | 26-32 37-41 49-62 93-98 105-115 160-168 195-207 214-222 271-277 299-308 315-322 348-357 377-384 403-416 431-444 481-497 506-512 553-570 |
|  | Antigenicity | 2-7 14-19 24-32 38-41 49-63 78-86 92-99 104-114 134-141 194-207 211-221 228-232 240-248 269-279 284-289 299-309 314-322 334-341 349-357 362-369 376-384 390-393 403-418 429-445 481-497 505-513 552-569 |
|  | Hydrophilicity | 1-5 25-32 50-65 94-111 135-140 150-159 195-208 214-219 243-248 269-276 301-306 315-320 334-342 350-369 374-383 386-396 406-416 423-443 481-498 505-517 552-570 |
| IEDB | Surface Accessibility | 25-31 49-62 93-98 195-207 269-274 350-356 373-378 389-394 429-435 481-490 505-514 551-571 |
|  | Flexibility | - |
|  | Antigenicity | 8-13 33-38 41-49 63-78 86-93 118-125 169-175 181-190 206-212 234-240 278-288 290-296 308-314 322-331 342-350 414-422 445-460 474-482 491-506 516-522 524-553 |
|  | Hydrophilicity | - |

| **Supplemental Table 4-3.** The predicted B-cell epitopes of Protein EmTSP3 by IEDB. | | | | | |
| --- | --- | --- | --- | --- | --- |
| Category | Serials | Amino acid sequence | Antigenicity | Allergenicity | Toxicity |
| LBEs | 18-28 | SSFSLVASWSD | 1.16 | Non-Allergen | Non-Toxin |
|  | 48-63 | CCGGVGPTDWSKPYPA | 0.93 | Non-Allergen | Non-Toxin |
|  | 66-80 | CKSGKENCTQPYQQG | 0.91 | Non-Allergen | Non-Toxin |
|  | 108-127 | IGAVICACCLAKKVNEYEKV | 0.31 | Non-Allergen | Non-Toxin |
|  | 22-33 | GGKNVTRSVGSF | 1.6 | Non-Allergen | Non-Toxin |
|  | 1-8 | VLLVYRHE | 0.9 | Probable-Allergen | Non-Toxin |
|  | 66-82 | NAFSLQNFTHSATSLSE | 1.21 | Non-Allergen | Non-Toxin |
| CBEs | - | A:K90, A:D91, A:S92, A:S93, A:L94, A:A95, A:G97, A:L98, A:L101, A:I102, A:L105, A:I108, A:G109, A:A110, A:V111, A:I112, A:C113, A:A114, A:C115, A:C116, A:L117, A:A118, A:K119, A:K120, A:V121, A:N122, A:E123, A:Y124, A:E125, A:K126 | 0.65 | Non-Allergen | Non-Toxin |
|  | - | A:D23, A:L24, A:T25, A:A26, A:H27, A:G28, A:R29, A:N30, A:A31, A:S32, A:D33, A:P34 | - | Probable-Allergen | Non-Toxin |
|  | - | A:C66, A:K67, A:S68, A:G69, A:K70 | - | Non-Allergen | Non-Toxin |
|  | - | A:V1, A:L2, A:L3, A:V4, A:Y5 | - | Non-Allergen | Non-Toxin |

*None of the latter three conformational epitopes showed detectable antigenicity

| **Supplemental Table 4-4.** The predicted B-cell epitopes of Protein EmTIP by IEDB. | | | | | |
| --- | --- | --- | --- | --- | --- |
| Category | Serials | Amino acid sequence | Antigenicity | Allergenicity | Toxicity |
| LBEs | 6-16 | ASWSDSSFSLV | -0.28 | Non-Allergen | Non-Toxin |
|  | 105-116 | VTRSVGSFGGKN | -0.19 | Non-Allergen | Non-Toxin |
|  | 149-165 | NFTHSATSLSENAFSLQ | 0.33 | Non-Allergen | Non-Toxin |
|  | 483-495 | TQQARKHKLTFIV | 1.37 | Non-Allergen | Non-Toxin |
|  | 553-569 | VRSDQKERMQESQRFHF | 1.03 | Non-Allergen | Non-Toxin |
|  | 313-326 | LKCTRSSGLRPIIL | 0.79 | Non-Allergen | Non-Toxin |
|  | 404-418 | TGRCGSPSQCPDGVL | 0.42 | Non-Allergen | Non-Toxin |
|  | 432-440 | ETQGADGGR | 0.76 | Non-Allergen | Non-Toxin |
| CBEs | - | A:E432, A:Q434, A:G435, A:A436, A:D437, A:G438, A:G439, A:R440, A:F468, A:Y471, A:E473, A:K490, A:T492, A:F493 | 1.83 | Non-Allergen | Non-Toxin |
|  | - | A:P49, A:N50, A:A51, A:K52, A:Q53, A:T54, A:D55, A:G56, A:K57, A:P58 | 1.25 | Non-Allergen | Non-Toxin |


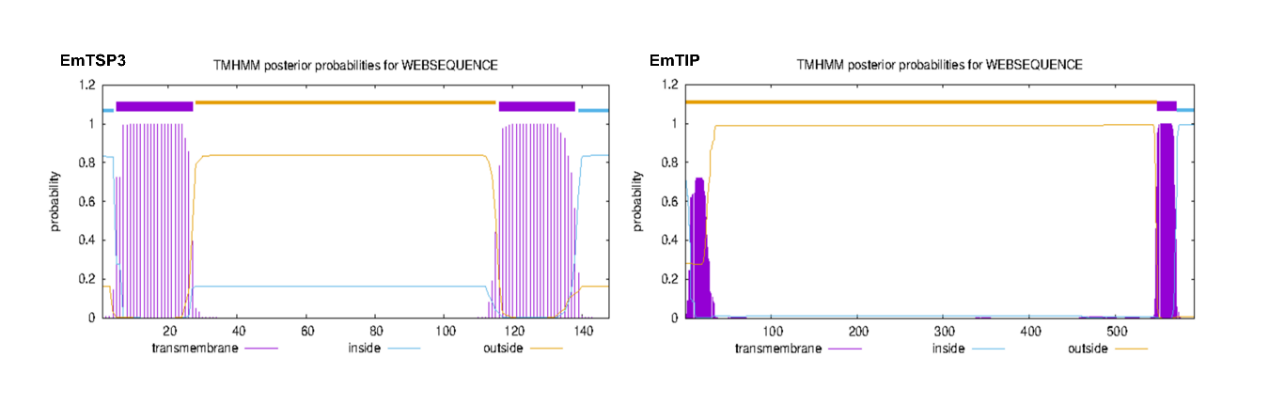


**Supplemental Figure 1.** The Protein EmTSP3 have 2 transmembrane domains and the Protein EmTIP had 1 transmembrane domain.


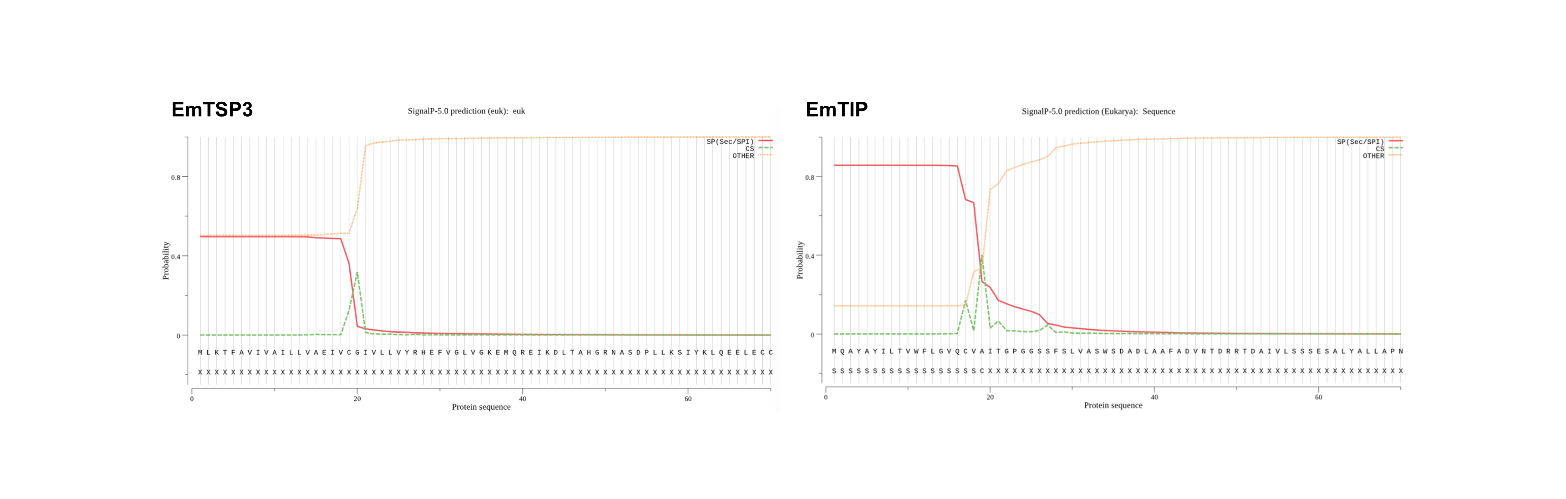


**Supplemental Figure 2.** Signal peptide of Protein EmTSP3 and EmTIP.


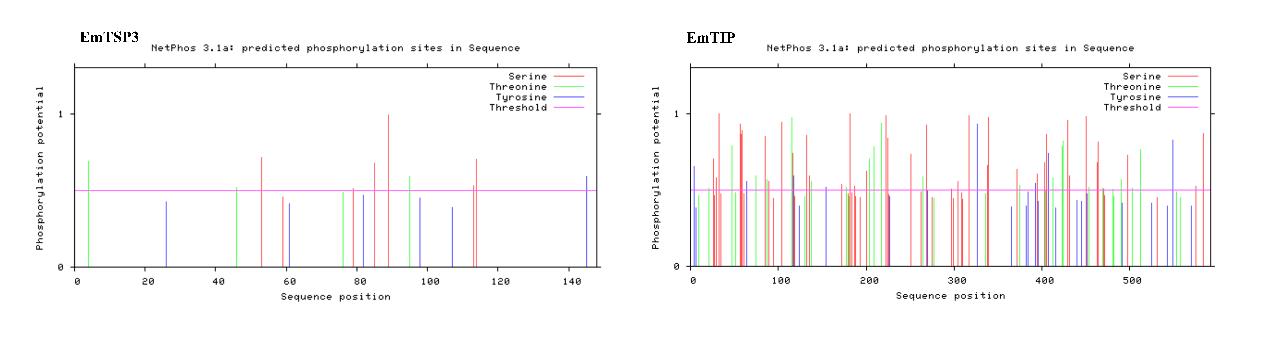
 **Supplemental Figure 3.** NetPhos 3.1 analysis showed Protein EmTSP3 has 6 serine, 3 threonine, and 9 tyrosine phosphorylation sites, mainly targeted by PKC, PKA, and CKI. Protein EmTIP has 39 serine, 21 threonine, and 5 tyrosine phosphorylation sites, mainly targeted by PKC, PKA, DNA-PK, and cDC2.


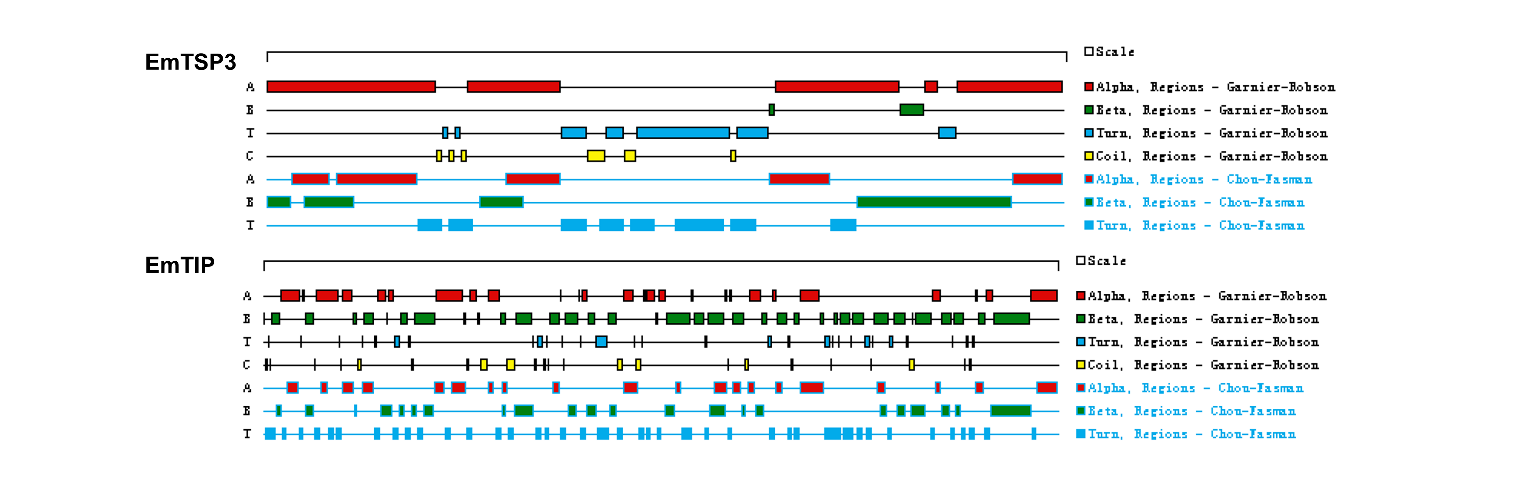


**Supplemental Figure 4.** The secondary structure prediction of Protein EmTSP3 and EmTIP was conducted using DNASTAR. The Gramier-Robson method, represented by lines A, B, T, and C, utilized colors to indicate different structural elements: α-helix (red), β-fold (green), β-turn (blue), and random coil (yellow). Additionally, the Chou-Fasman method employed lines A, E, and T to depict the same structural elements without including a random coil structure.


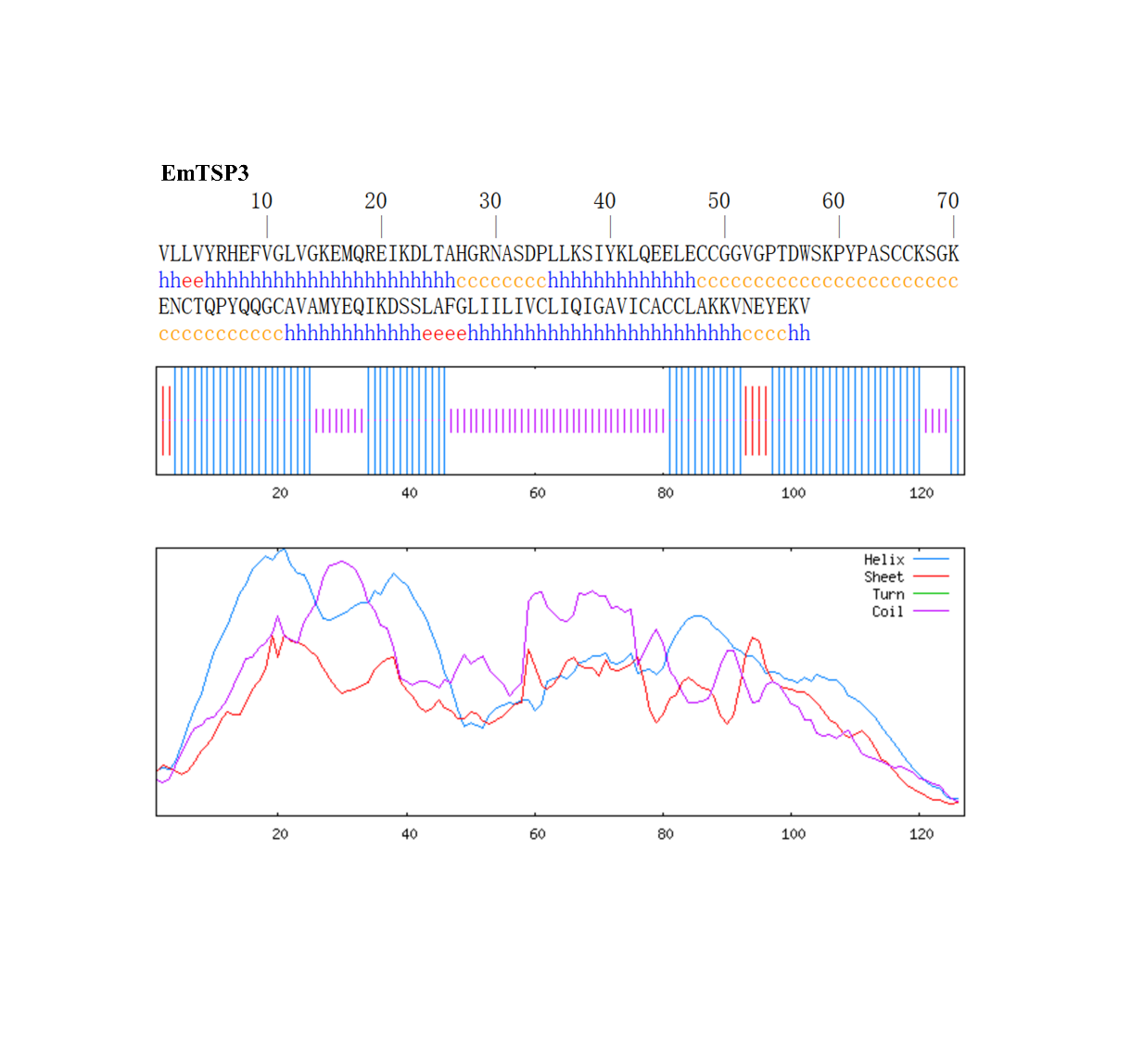


**Supplemental Figure 5-1.** Secondary structure of Protein EmTSP3 by SOPMA. The result of server
SOPMA shows that the blue h is Alpha helix, the red e is Extended strand, the green t is Beta turn, the yellow c is Random coil. Protein EmTSP3 consisted of 60.81% Alpha helix, 9.46% Extended strand, 2.7% Beta turn, and 27.03% Random coil structures.
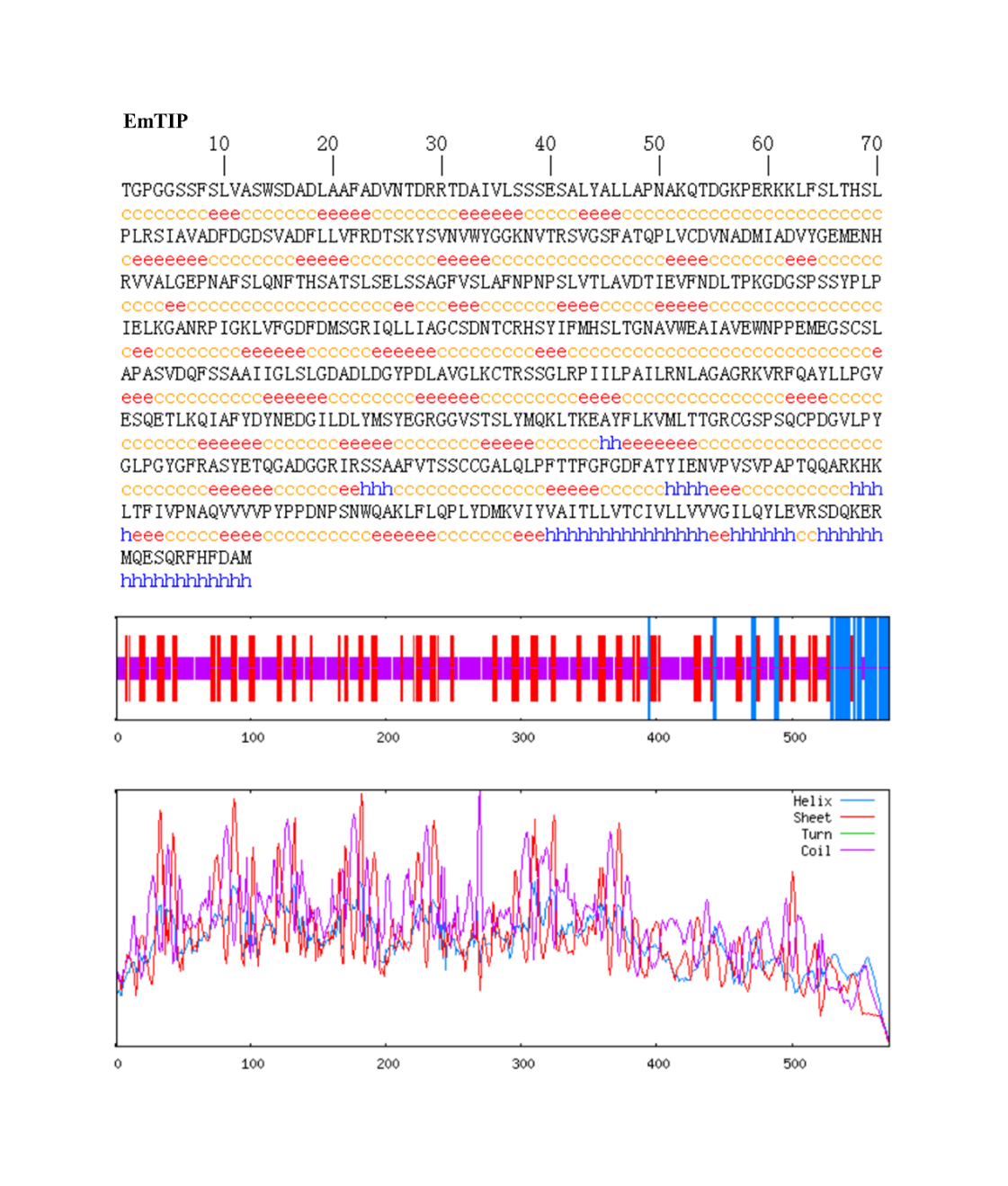


**Supplemental Figure 5-2.** Secondary structure of Protein EmTIP by SOPMA. The result of server SOPMA shows that the blue h is Alpha helix, the red e is Extended strand, the green t is Beta turn, the yellow c is Random coil. Protein EmTIP consisted of 15.71% Alpha helix, 30.07% Extended strand, 3.21% Beta turn, and 51.01% Random coil structures


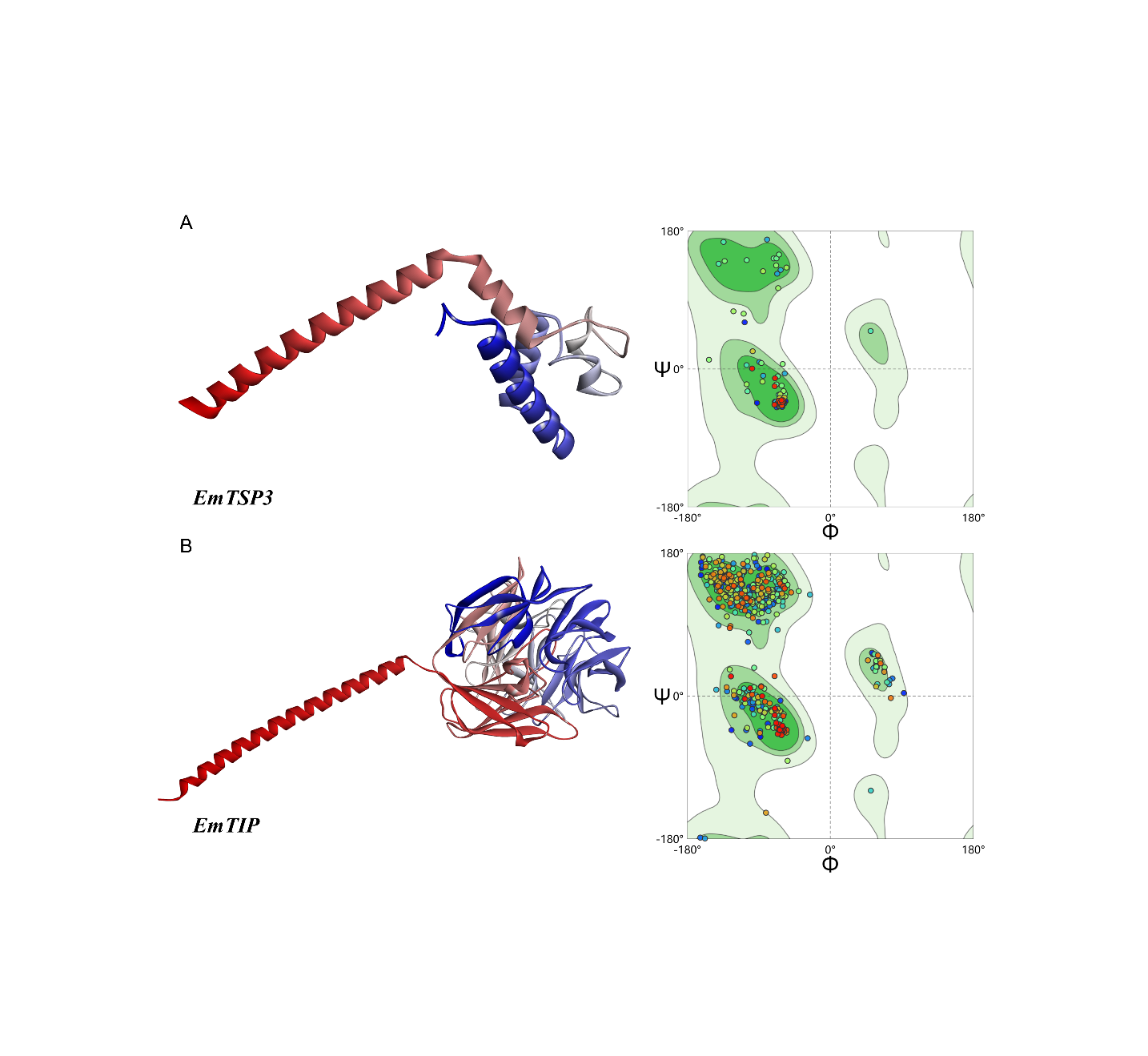


**Supplemental Figure 6.** (A) Tertiary structures of Protein EmTSP3. (B) Tertiary structures of Protein EmTIP. Next to each structure is a Ramachandran plot indicating allowed and disallowed rotation angles of peptide bonds. Inside the dark green circle represents completely allowed areas, inside the light green circle represents allowed areas, and outside the green circle is not allowed. A good model has over 90% of scatter within these circles.


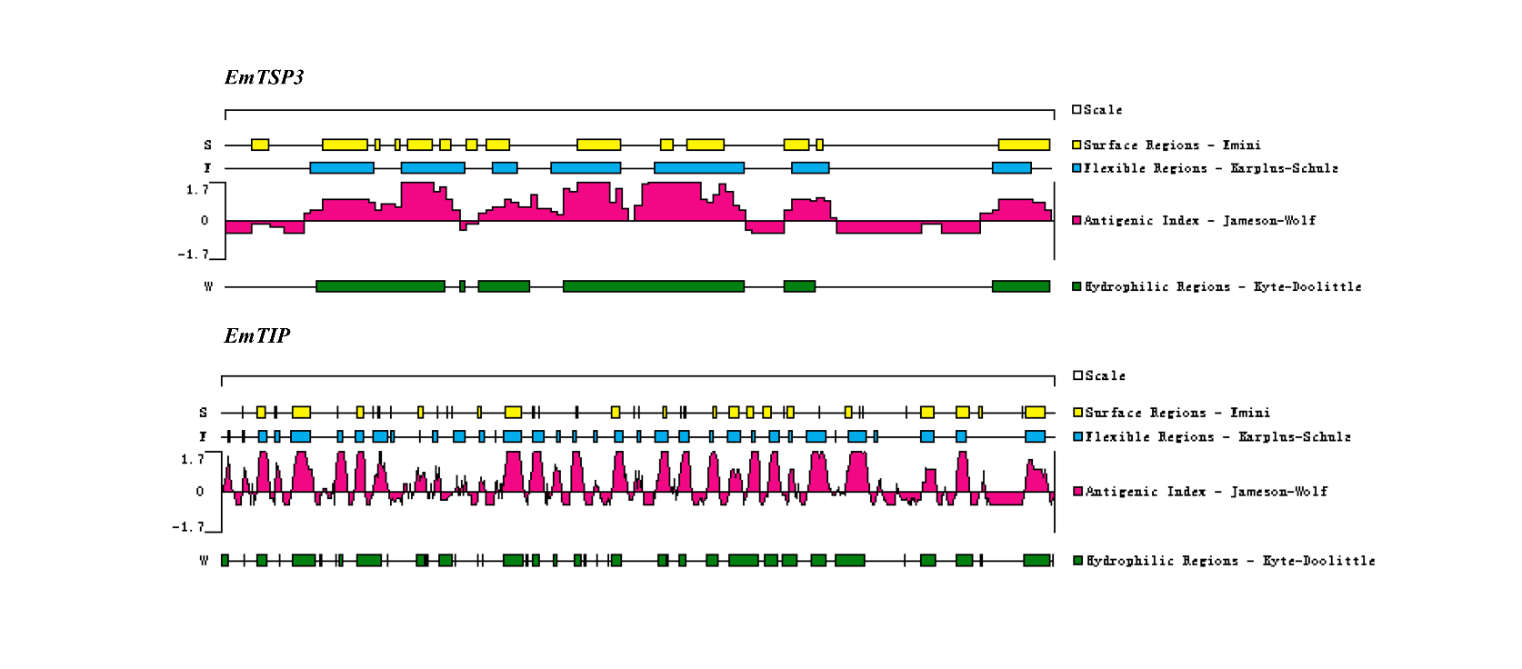


**Supplemental Figures 7.** B-cell epitopes of Protein EmTSP3 and EmTIP by DNASTAR. The yellow area represents Surface accessibility, the blue area represents Flexibility, the pink area represents Antigenicity and the green area represents Hydrophobicity. We need to obtain the areas above the threshold of the purple area, and the rectangles on the other three lines.


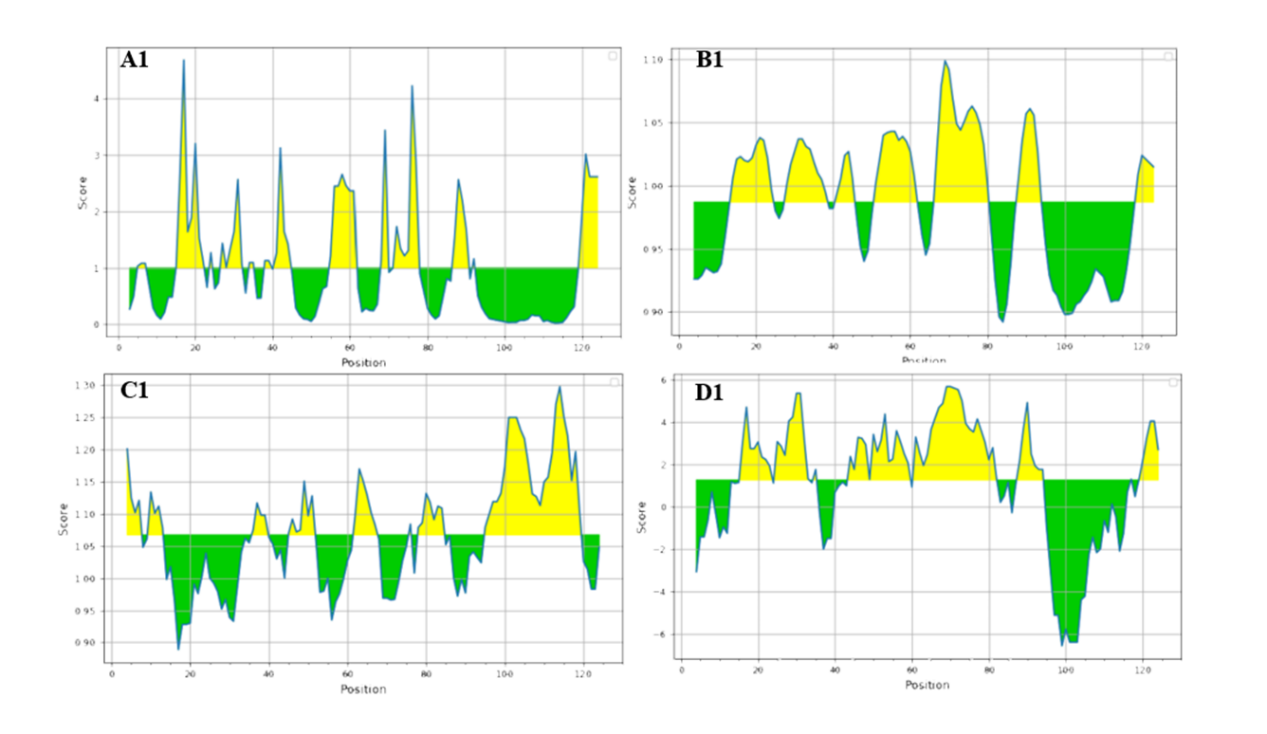


**Supplemental Figures 8-1.** B-cell epitopes of Protein EmTSP3 by IEDB. The A1 is Surface accessibility, the B1 is Flexibility, the C1 is Antigenicity, the D1 is Hydrophilicity. The yellow area represents primary result.


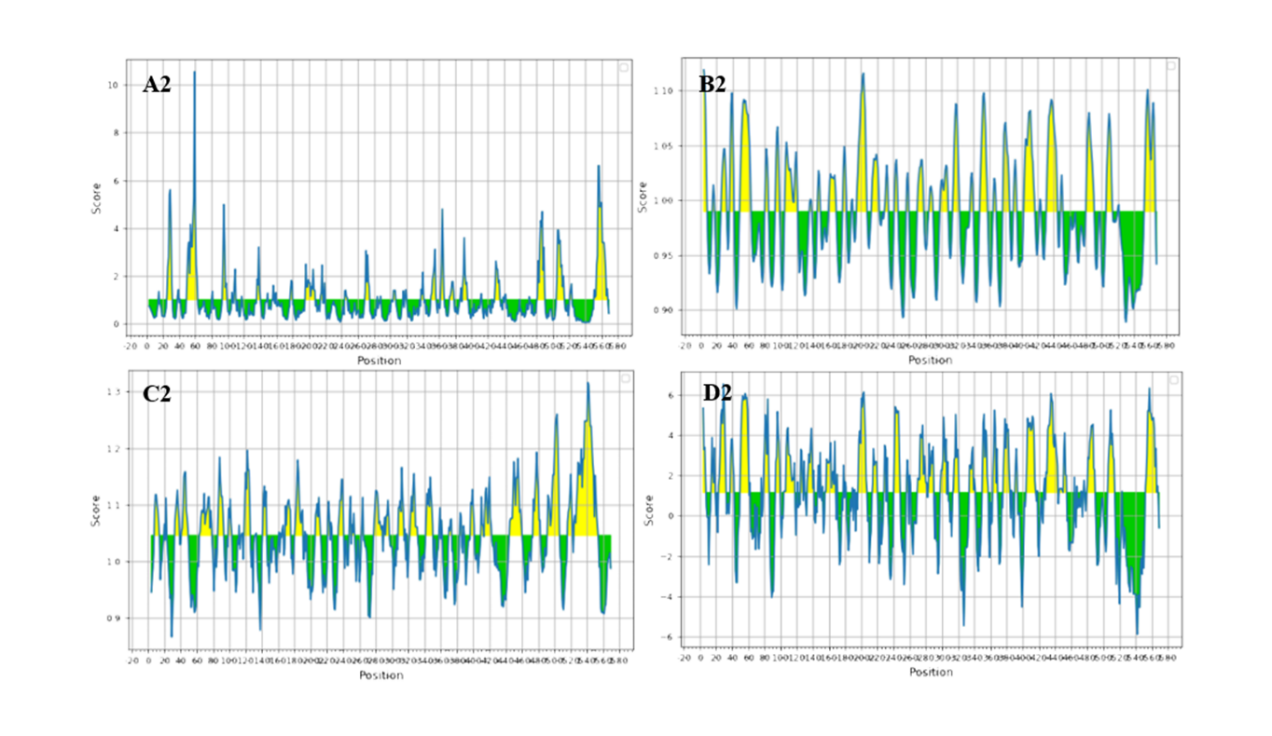


**Supplemental Figures 8-2.** B-cell epitopes of Protein EmTIP by IEDB. The A2 is Surface accessibility, the B2 is Flexibility, the C2 is Antigenicity, the D2 is Hydrophilicity. The yellow area represents primary result.


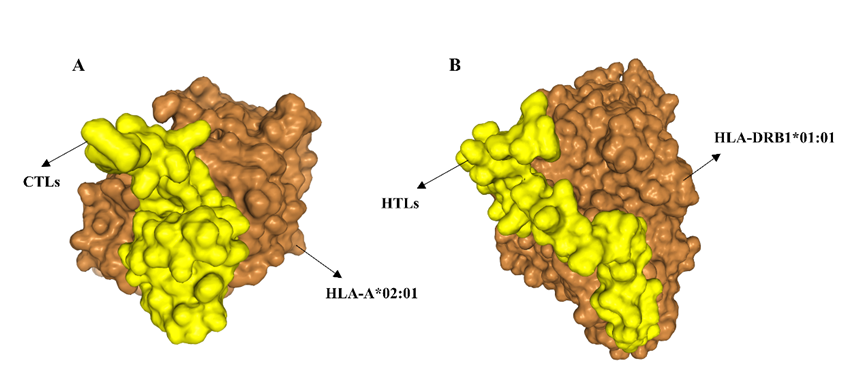


**Supplemental Figure 9.** Results of the HLA-peptide complexes. (A) HLA-A*02:01 and CTL epitopes docking result. (B) HLA-DRB1*01:01 and HTL epitopes docking result.


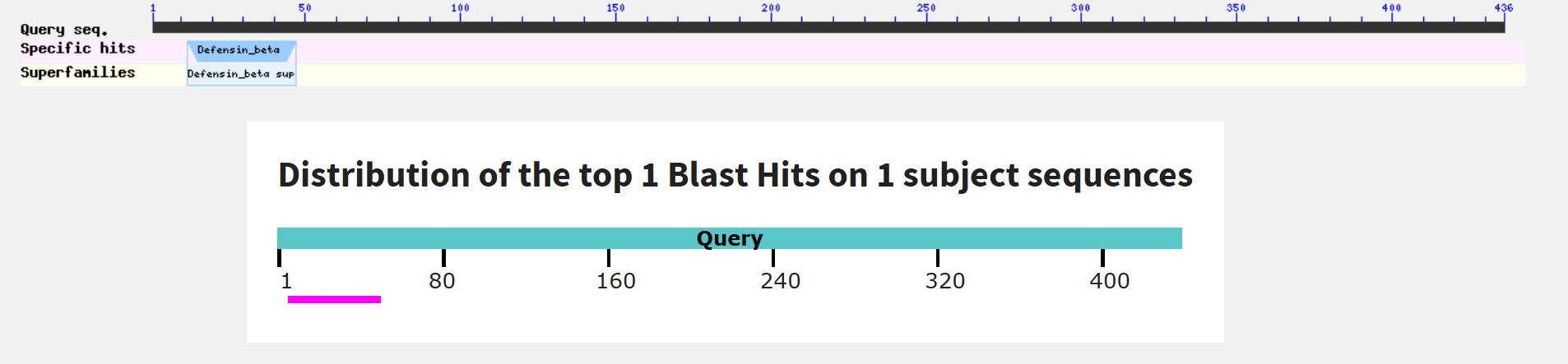


**Supplemental Figure 10.** The top chart illustrates the length of the query sequence, approximately 430 amino acids or nucleotides. A matching region at the beginning (positions 1-80) is highlighted by a pink bar. This indicates high similarity to the known Beta-defensin 3 protein at the beginning, covering only about 10% of the entire sequence.


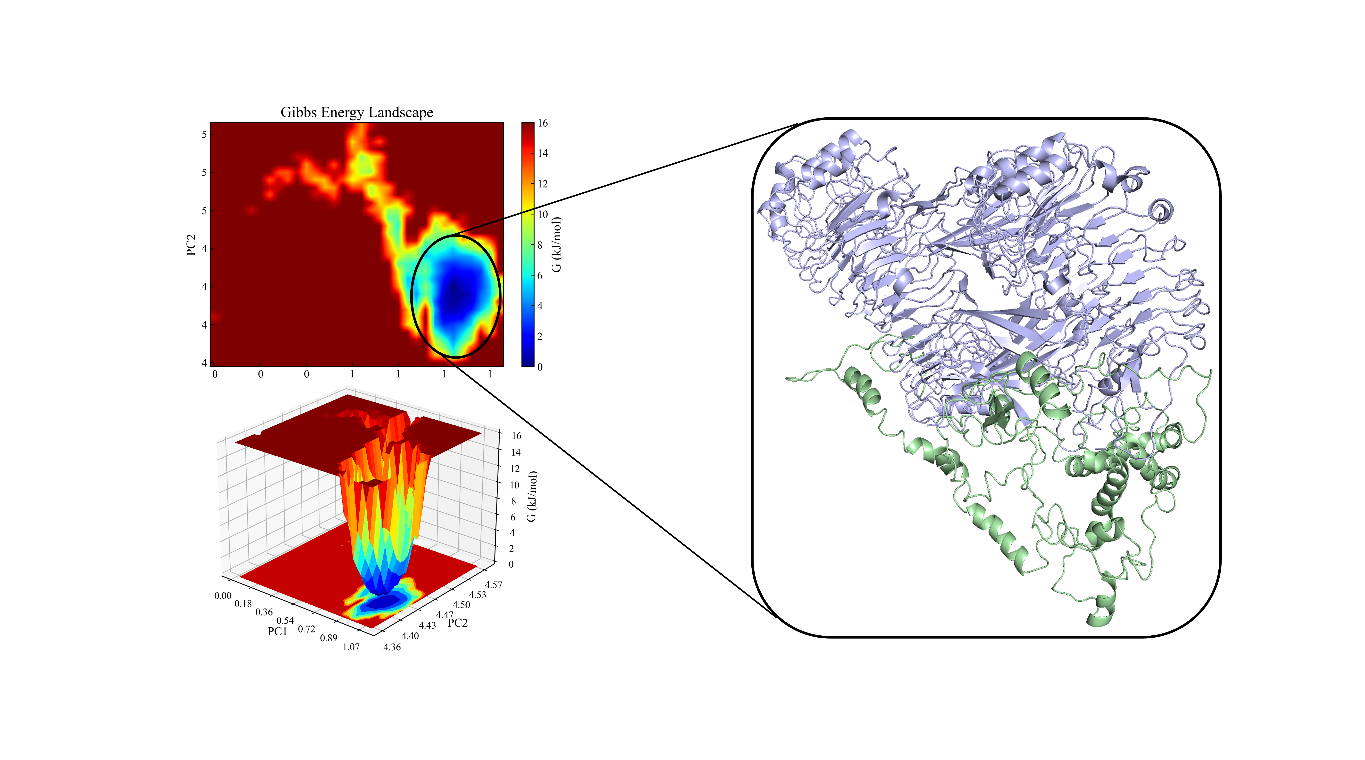


**Supplemental Figure 11.** Protein conformation at the low point of the Free Energy Landscape.


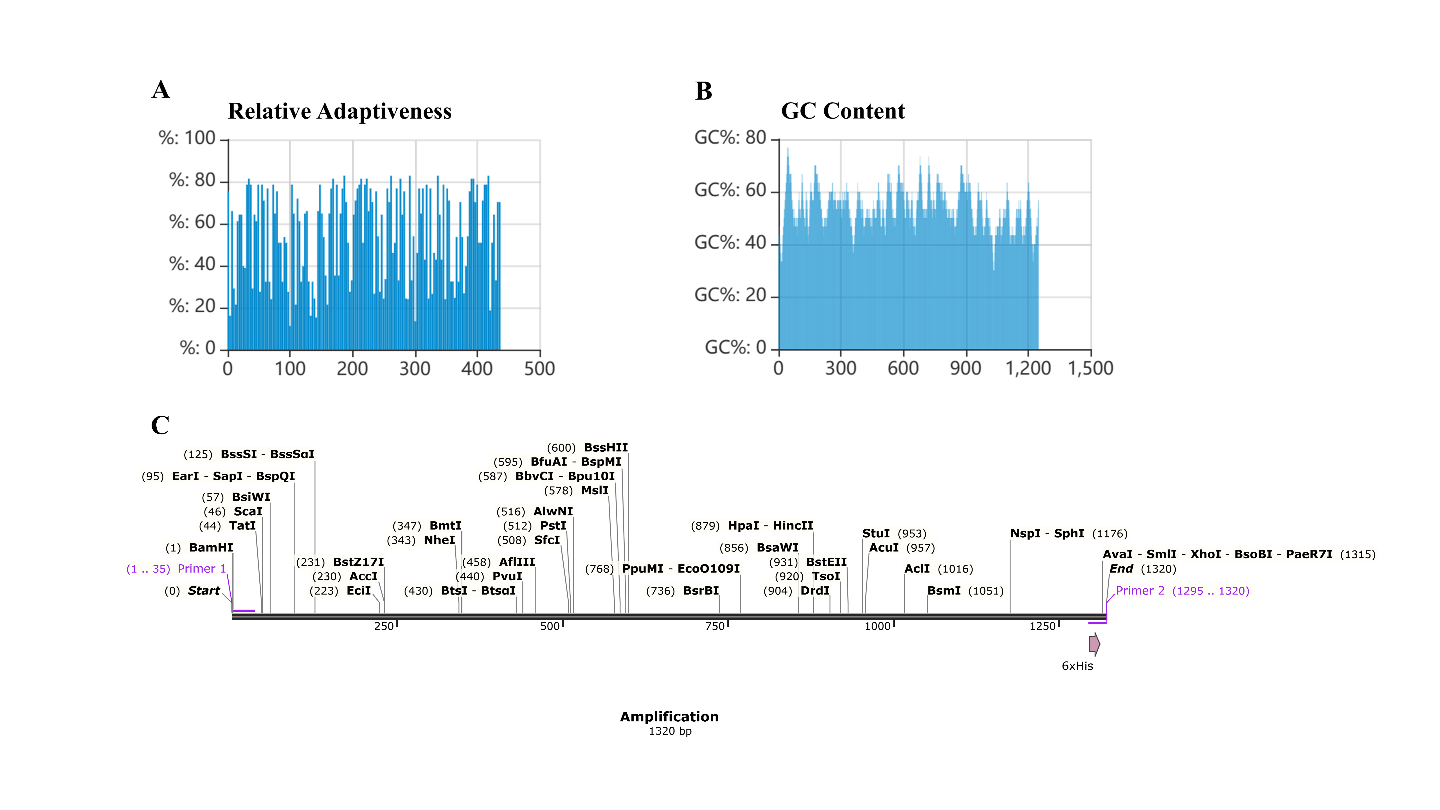


**Supplemental Figure 12.** (A) Adaptiveness of optimized codon. (B) The GC content. (C) The MEV (1320 bp) after amplified.
